# Tracking tsunami propagation and Island’s collapse after the Hunga Tonga Hunga Ha’apai 2022 volcanic eruption from multi-space observations

**DOI:** 10.1038/s41598-023-46397-1

**Published:** 2023-11-17

**Authors:** Mahesh N. Shrivastava, A. S. Sunil, Ajeet K. Maurya, Felipe Aguilera, Simón Orrego, P. S. Sunil, Rodrigo Cienfuegos, Marcos Moreno

**Affiliations:** 1 Departamento de Ciencias Geologicas, Universidad Católica del Norte, Antofagasta, Chile; 2 Millenium Institute on Volcanic Risk Research - Ckelar Volcanoes, Antofagasta, Chile; 3 Centro de Investigación para la Gestión Integrada del Riesgo de Desastres, Santiago, Chile; 4 Department of Marine Geology and Geophysics, School of Marine Sciences, Cochin University of Science and Technology, Kochi, India; 5 Department of Physics, Babasaheb Bhimrao Ambedkar University, Lucknow, India; 6 Departamento de Ingeniería Hidráulica y Ambiental, Pontificia Universidad Católica de Chile, Santiago, Chile; 7 Departmento de Ingeniería Estructural y Geotécnica, Pontificia Universidad Católica de Chile, Santiago, Chile

**Keywords:** Volcanology, Natural hazards, Space physics

## Abstract

The quantity and accuracy of satellite-geodetic measurements have increased over time, revolutionizing the monitoring of tectonic processes. Global Navigation Satellite System (GNSS) and satellite radar signals provide observations beyond ground deformation, including how earthquake and tsunami processes affect variations in the ionosphere. Here, we study the Hunga Tonga Hunga Ha’apai (HTHH) volcanic eruption 2022 and its associated tsunami propagation with the analysis GNSS derived Total Electron Content (TEC), Synthetic Aperture Radar (SAR) Sentinel-1 data, complemented with tide gauge observations. We utilize GNSS sites data within a ~ 5000 km radius from the volcanic eruption for estimating the ionospheric perturbation as Vertical TEC. We give evidence on the detection of acoustic gravity, internal gravity, and atmospheric Lamb waves signatures in the TEC perturbation. In particular, the internal gravity waves that concentrated in the southwest of Tonga, directly correlates with the observed tsunami propagation direction as accounted by the tide gauge measurements. However, the acoustic gravity wave signature in the TEC is dominant in the north direction suggesting a surface deformation, which could be verified using Sentinel-1A SAR amplitude data. The analysis presented herein shows that within 5 h of the volcanic eruption, the central part of the HTHH island landscape disappeared with the biggest explosion. The unprecedented detail resolved by integrating satellite data yields previously unknown details of the deformation of the 2022 HTHH volcano eruption.

## Introduction

In addition to the conventional three-dimensional space, simultaneous multi-satellite geodetic monitoring provides four-dimensional mapping, where time is becoming critical in order to map non steady state processes of the solid earth with better spatial resolution and accuracy. Geodesy is currently concerned with the physics of the earth, oceans, atmosphere, and cryosphere across all scales, in addition to assisting global surveying and mapping. This has led to cutting edge analysis combining several data sets to examine the process of natural hazards^1,2^. In modern seismology, the GNSS and Interferometry assessed from SAR observations are widely used to understand the accumulation of the seismic energy during the inter-seismic period and release of the energy during the co-seismic period in the form of an earthquake^3,4^. It has also been documented that surface deformation due to earthquakes, tsunamis and volcanic eruptions produces ionospheric changes in the TEC^5–10^. These changes can be detected by GNSS instrumentations, which have led to new observations capabilities for monitoring the coupling processes between Lithosphere and Ionosphere^5,10,11^.

The ionosphere is an ionized part of the Earth’s atmosphere and is confined with in an altitude of approximately 60–1000 km. It has ionized gas, called plasma, which influences radio wave propagation^12^. The ionosphere expands and shrinks depending on the energy it exchanges from the top sources (e.g., the sun, and solar activity) and the bottom sources (e.g., tropospheric and lithospheric sources). The lithospheric disturbances are predominantly caused by natural sources (e.g., earthquakes, volcanic eruptions, tsunami and cryospheric changes such as mountain glaciers, continental ice sheets, seasonal snow ice cover on land and sea ice)^10,13–16^ or human activity (e.g., nuclear explosions)^17^. The ionosphere can be perturbed with several types of waves, which may be excited by volcanic eruptions, earthquakes, and tsunamis (e.g., acoustic gravity wave, internal gravity wave^18^, acoustic Rayleigh wave, and atmospheric Lamb wave^7^). The acoustic gravity wave generates at the epicentral area is enhanced by the vertical displacement of the ground/ocean, which contains high‐frequency signal components (0.5–1.0 Hz). This wave propagates with speed of around 500–1000 m/s and appear in the ionosphere after nearly 8 mins^19,20^. This wave frequency is higher than the Brunt-Väissälä frequency ~ 2.9 mHz. The internal gravity wave are generated with the tsunami propagation. The frequency of this wave is lower than the Brunt-Väissälä frequency ~ 2.9 mHz and propagates with speed of around 200–300 m/s. The acoustic Rayleigh wave is the acoustic wave coupled with the seismic Rayleigh wave. This wave has recognized in two main frequencies of 3.68 mHz and 4.44 mHz, and propagation velocities ~ 2.2 and 3.5 km/s respectively^21^. The atmospheric Lamb wave mostly observed during the volcanic eruption that travels at the sound speed, typically 300–350 m/s in the troposphere and can exist at any frequencies. Although their energy is confined to the troposphere, their amplitudes increase exponentially with height due to decreasing atmospheric density. Their wave energy can leak into the upper atmosphere when Lamb waves horizontal phase speed is resonant with the atmosphere, as can be the case with acoustic gravity and internal gravity waves^22–24^. Atmospheric Lamb waves with ~ 319 m/s phase speed were previously identified as an atmospheric wave response to the Krakatoa eruption^25,26^.

The HTHH volcano includes two small islands along a submarine caldera in the western South Pacific Ocean^27^. The underwater volcanoes in this region stand above a massive tectonic collision zone, where the western edge of the Pacific tectonic plate sinks beneath the Indo–Australian plate^27^. The edge of the Pacific plate heats up as it sinks into the molten rock that rises to feed the volcanoes of the Tonga–Kermadec arc. The biggest HTHH volcanic eruption, the largest event of its type in the last three decades, occurred on the sea surface on January 15, 2022 along the Caldera rim^28^. This volcanic eruption occurred in the southern portion of the volcano which was active during the 2014–2015 eruption^27^. The 2014–2015 volcanic eruption started around December 19, 2014, when a Surtseyan volcanic eruption was observed near 20.5° S. 175.4° W in the Tonga-Kermadec Islands volcanic arc^29^, followed by the emergence of a new island HTHH by early 2015^30^. The HTHH volcanic eruption 2022 had a massive impact by ejecting a huge volume of ashes and volcanic gasses in the atmosphere and generated a significant unleashing tsunami that affected locally over Tango’s nearby island and many Pacific rim countries from South America, North America, Alaska, and Japan^27^. The volcanic explosion released an enormous ash plume well recorded by satellite imaging. The plume rising above HTHH significantly affected the Tonga islands generated an extreme atmospheric pressure wave towards high altitudes, which was recorded by meteorological and infrasound sensors across the World^31^.

Many recent studies have addressed the atmospheric and ionospheric impacts of HTHH volcanic eruptions^32,33^. Astafyeva et al.^32^ investigated the nearfield ionospheric total electron content variations associated with this event and estimated the amount of energy released during the major explosion. Using TEC measurements from globally distributed GNSS receivers, Themens et al.^33^ identified large scale and medium scale travelling ionospheric disturbances propagating away from the eruption site. Matoza et al.^34^ also detected explosive eruption that provoked different atmospheric wave modes such as infrasound, Lamb, and internal gravity waves traversing from ∼ 1 h period until audible, propagated at global ranges and reached the upper atmosphere layers. Apart from the ionospheric disturbances, HTHH volcanic eruption generated tsunami waves in the near and far-field which are reported in few recent studies^35–38^. Lynett et al.^39^, Carvajal et al.^35^, and Wright et al.^40^ reported that the volcano induced tsunami waves were detected around the Pacific coasts. Shrivastava et al.^41^ studied the Tonga volcanic eruption 2022 and its associated tsunami with GNSS derived TEC perturbation. Ravanelli et al.^42^ studied the ocean-ionospheric perturbation due to the 2022 Hunga Tonga Hunga‐Ha’apai Eruption. The plume of the larger HTHH volcanic eruption 2022 perturbed the sea surface and generated tsunami waves across the Pacific Ocean, even triggering atmospheric shock waves. These signals can be identified through GNSS-derived TEC changes obtained from the available GNSS sites^10,43–48^. The internal-gravity and acoustic gravity waves identified in the TEC variations can be used to infer the tsunami propagation directions and surface deformation anomalies^9,10^. The analysis and identification of these volcano-ionospheric signals provide new opportunities for remote sensing techniques, which may help to understand the magma propagation on the surface at the volcanic vent and the main characteristics of the generated tsunami waves^10,16^. In the present study, we utilize the GNSS derived TEC perturbation together with the analysis of Sentinel-1 SAR data to characterize the tsunami propagation and surface deformation timing associated with January 15, 2022, HTHH volcanic eruption.

### TEC perturbation during the HTHH volcanic eruption

**Figure 1:**
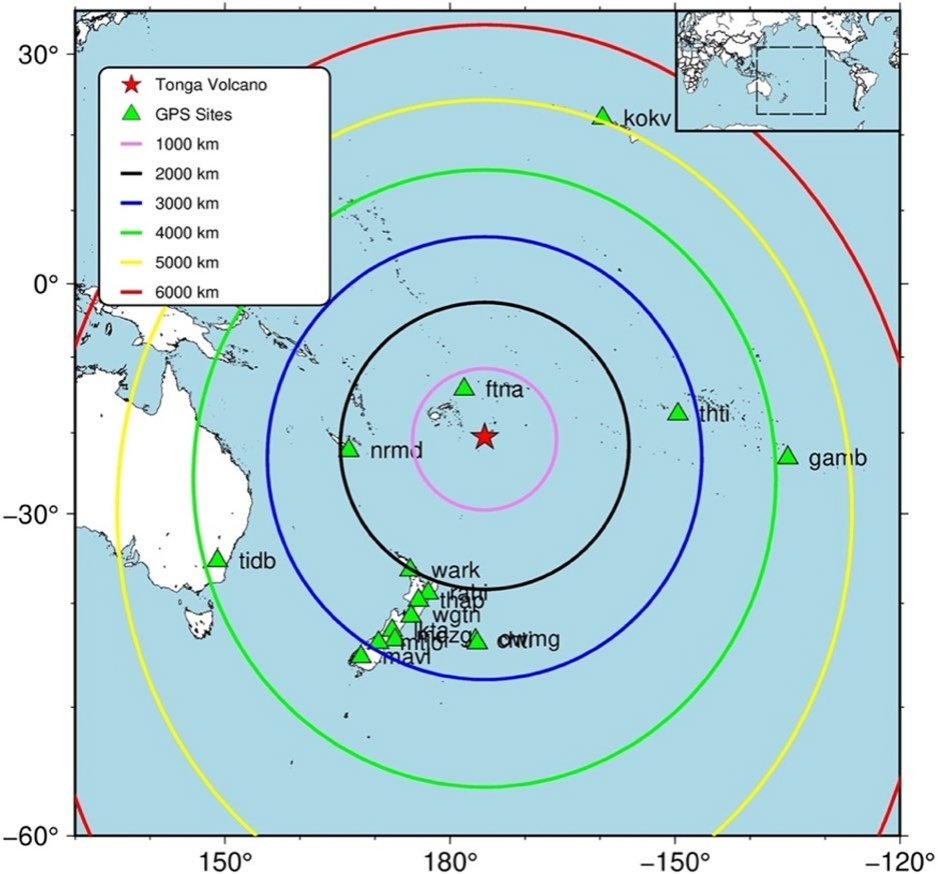
Geographic map of the island of Hunga Tonga and Hunga Ha’apai. Green triangles represent the locations of the GNSS sites that are used for the TEC perturbation study. The closed colored contour lines show the radial distance from the HTHH volcanic eruption vent (red filled star). The star represents the Hunga Tonga and Hunga Ha’apai volcanic eruption vent. This Figure was created using the Generic Mapping Tools (GMT) version 6.0.0 (https://www.generic-mapping-tools.org).

**Figure 2:**
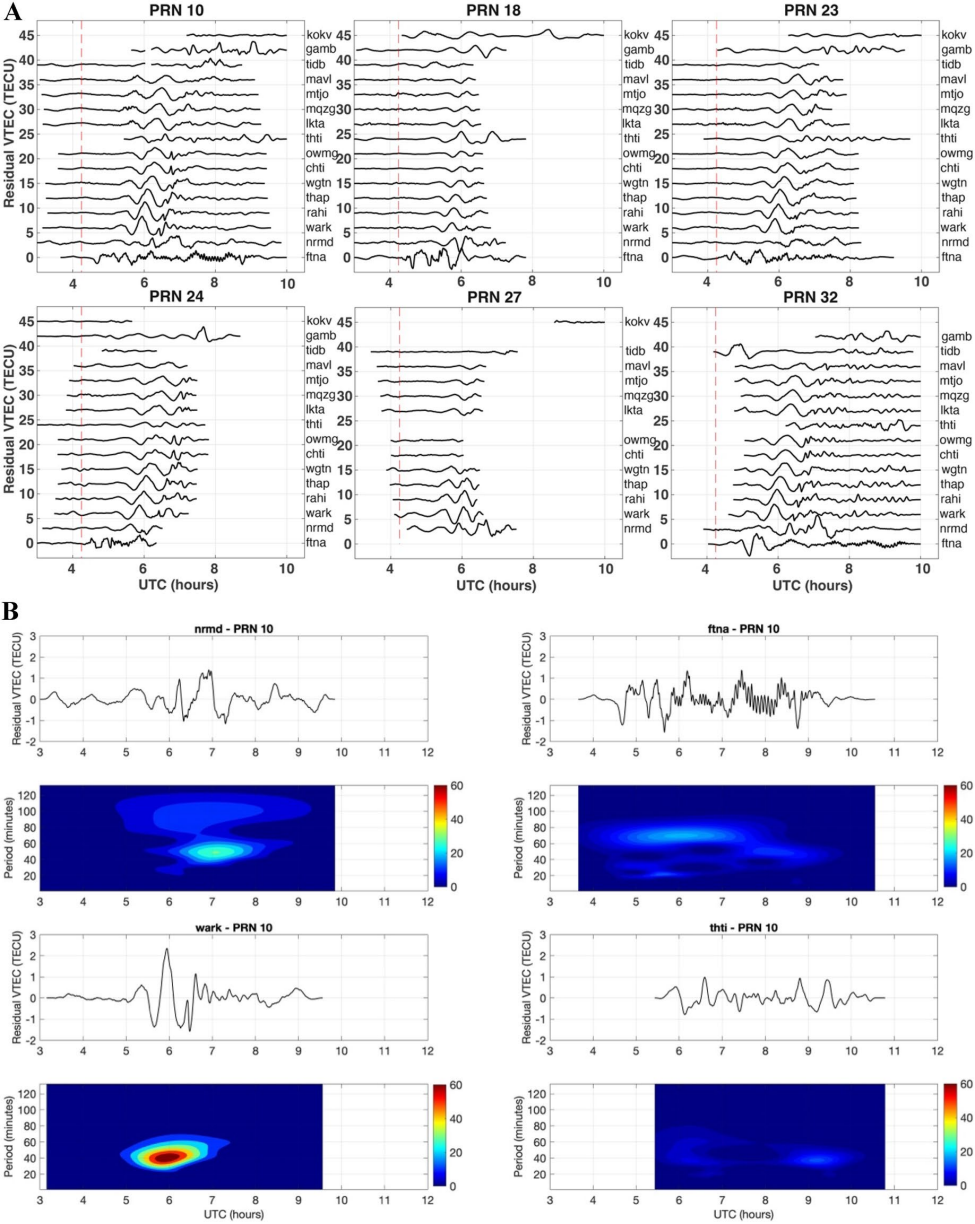

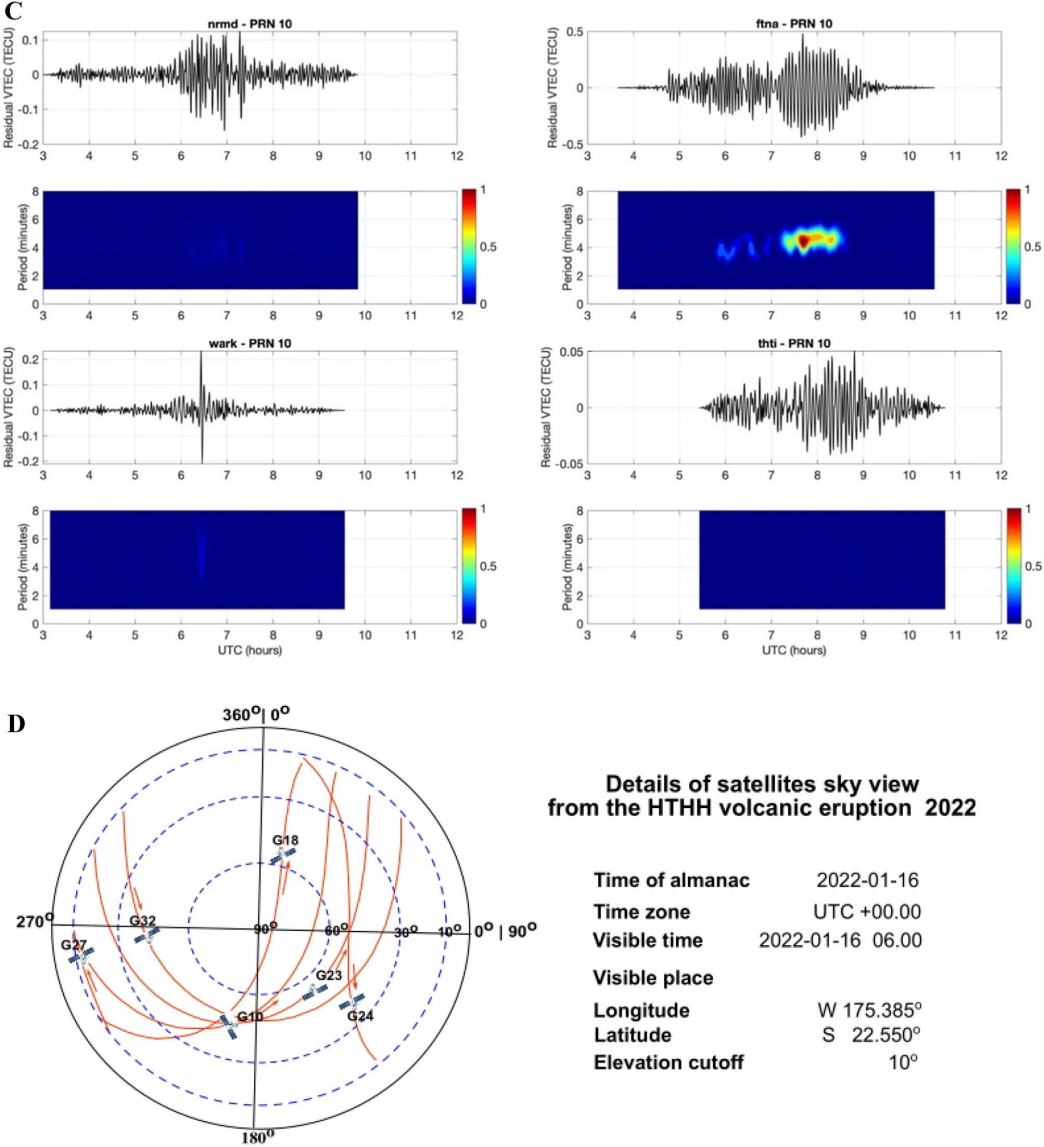

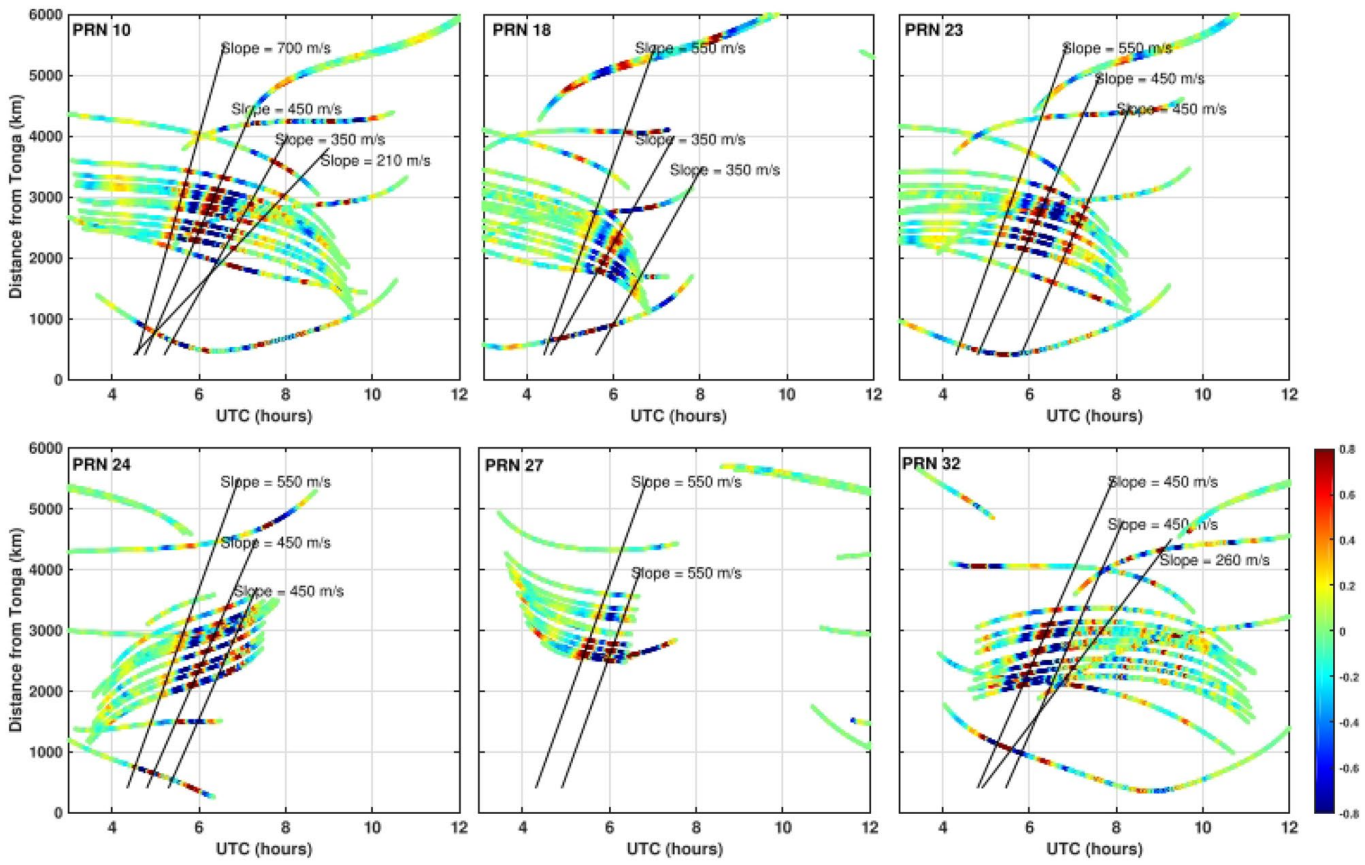
(**A**) The residual VTEC is presented for PRNs 10, 18, 23, 24, 27 and 32 during the HTHH volcanic eruption for the closest GNSS site ftna. The dotted red line marks the time of the volcanic eruption. The lines represent the residual VTEC regarding the locations of GNSS sites from closest to farthest. This Figure was created using the Generic Mapping Tools (GMT) version 6.0.0 (https://www.generic-mapping-tools.org). (**B**) The internal gravity waves signature in the spectrogram of the residual TEC changes at randomly chosen GNSS sites nrmd, ftna, thti and wark from west to south for PRNs 10 for the HTHH volcanic eruption. This Figure was created using the Generic Mapping Tools (GMT) version 6.0.0 (https://www.generic-mapping-tools.org). (**C**) The acoustic gravity waves signature in the spectrogram of the residual TEC changes at randomly chosen GNSS sites nrmd, ftna, thti and wark from west to south for PRNs 10 for the HTHH volcanic eruption. This Figure was created using the Generic Mapping Tools (GMT) version 6.0.0 (https://www.generic-mapping-tools.org). (**D**) The sky view of the GNSS satellites from the vent of the HTHH volcanic eruption. The GNSS satellites are referred as G and PRN. Hodochrone map of PRN 10, 18, 23, 24, 27, and 32. The residual VTEC are plotted as a function of distance from HTHH volcano and time. This Figure was created using the Generic Mapping Tools (GMT) version 6.0.0 (https://www.generic-mapping-tools.org).

To investigate the ionospheric perturbations associated with the HTHH volcanic eruption region around 6000 km, we have analyzed Vertical TEC (VTEC) data from 16 continuous GNSS sites (Fig. 1). The arrival times of the TEC perturbations appears at each GNSS site with respect to distance from the HTHH volcanic eruption. The VTEC data from satellite Pseudo-Random Numbers (PRNs) 10, 18, 23, 24, 27, and 32 are used to identify the eruption signatures in ionosphere. To remove the long periodicities and to extract the eruption signatures in VTEC, we have applied a band pass filter with cut-off frequencies between 0.2 and 10 mHz. Figure 2A depicts the residual VTEC variations during the period between 2 and 12 UTC. In the Fig. 1, the nearest GNSS site ftna shows significant VTEC variations in PRN 10, 18, 23, 24, 27 and 32. Apart from that, the presence of a particular perturbation which lasts for a longer duration is very clear in PRN 10 and 32. In Fig. 2A, the residual VTEC from all the GNSS sites are arranged as a function of distance from HTHH volcano. The presence of significant VTEC perturbations in all the PRNs with a gradual time delay as moving away from the volcano is clearly seen in Fig. 2A. These PRNs exhibit significant TEC changes of approximately 2.5 TECU near to the HTHH volcanic eruption region (Fig. 2A). Interestingly, the TEC variations observed at the closest GNSS site ftna detected by PRNs 10, 23, 24, and 32 at elevations ranging from 30° to 60° show a wavy signal characteristic (Fig. 2A). The sub-ionospheric point (SIP) tracks of PRN’s 10, 18, 23, 24, 27 and 32 at GNSS sites are provided in supplementary information in Fig. S1.

Since the VTEC is highly variable and perturbed by space weather (solar flare and geomagnetic storm) conditions^49,50^, we have checked the space weather condition from (https://www.spaceweatherlive.com/) and confirmed that there was no solar flare activity took place during January 15–18, 2022. We have also checked three hourly planetary K index (K_p_) and disturbance storm time index (Dst) providing the conditions for the geomagnetic activity (https://wdc.kugi.kyoto-u.ac.jp/) during the same time window. The daily Kp index was in the range 2.0–3.3 during this period. The Dst index shows a moderate geomagnetic storm with minimum Dst ~ 91 nT around 23 UTC on January 14, 2022, which was recovered on January 15, 2022.

The residual VTEC (Fig. 2A) signals indicate the presence of waves with different periodicity components. To distinguish them, we apply two types of band-pass filters. In the lower atmosphere, the acoustic gravity cut-off frequency and Brunt-Väissälä frequencies are 3.3 mHz (~ 5.05 min) and 2.9 mHz (~ 5.75 min) respectively^47,48^. The waves with frequencies higher than acoustic gravity cut-off frequency are considered as acoustic gravity waves and lower than Brunt-Väissälä frequency are treated as internal gravity waves. Therefore, to extract the acoustic gravity wave signatures, a second order band pass filter is applied with cut-off frequencies between 3 and 10 mHz. Similarly, a cut-off frequency range of 0.2–10 mHz is used for the internal gravity wave signature. We have also performed the wavelet analysis of the filtered VTEC data using the Morlet wavelet analysis technique^51,52^ to appraise the temporal evolution of the frequency content of the signal. The Morlet wavelet analysis for internal gravity and acoustic gravity wave signatures at the four closest GNSS sites nrmd, ftna, thti, and wark using VTEC data of PRN 10 are presented in Fig. 2B,C. The internal gravity wave signature for all the GNSS sites is presented in the supplementary information as Fig. S2, and the acoustic gravity wave signature for all the GNSS sites is presented as Fig. S3. From the Fig. 2B, internal gravity wave signatures are more dominant in GNSS sites wark and nrmd, which are located south and west of the HTHH volcano respectively. On the other hand, maximum power of eruption induced acoustic gravity waves are observed in ftna, which is a GNSS site on the north side of the eruption.

The acoustic Rayleigh and atmospheric Lamb waves are not easily distinguishable in the spectrogram due to same periodicity. It is necessary to further analyze these waves in ionospheric imprints of HTHH volcano eruption. We have generated the hodochrone map from the residual VTECs of PRN 10, 18, 23, 24, 27, and 32 (Fig. 2D). The PRN 10 and 32 could be observe residual VTEC longer than other PRNs. Therefore, these PRN’s detected acoustic gravity with two velocities in the range 450–700 m/s, atmospheric Lamb wave with velocity 350 m/s. The GNSS PRN 10 and PRN 32 were in the south west of the vent of HTHH volcanic eruption sensed internal gravity wave velocities 210 m/s and 260 m/s respectively. Other PRN’s detects the acoustic gravity wave with velocity in the range of 450–550 m/s and atmospheric Lamb wave with velocity 350 m/s.

### Changes in HTHH volcanic eruption morphology detected by SAR data

**Figure 3:**
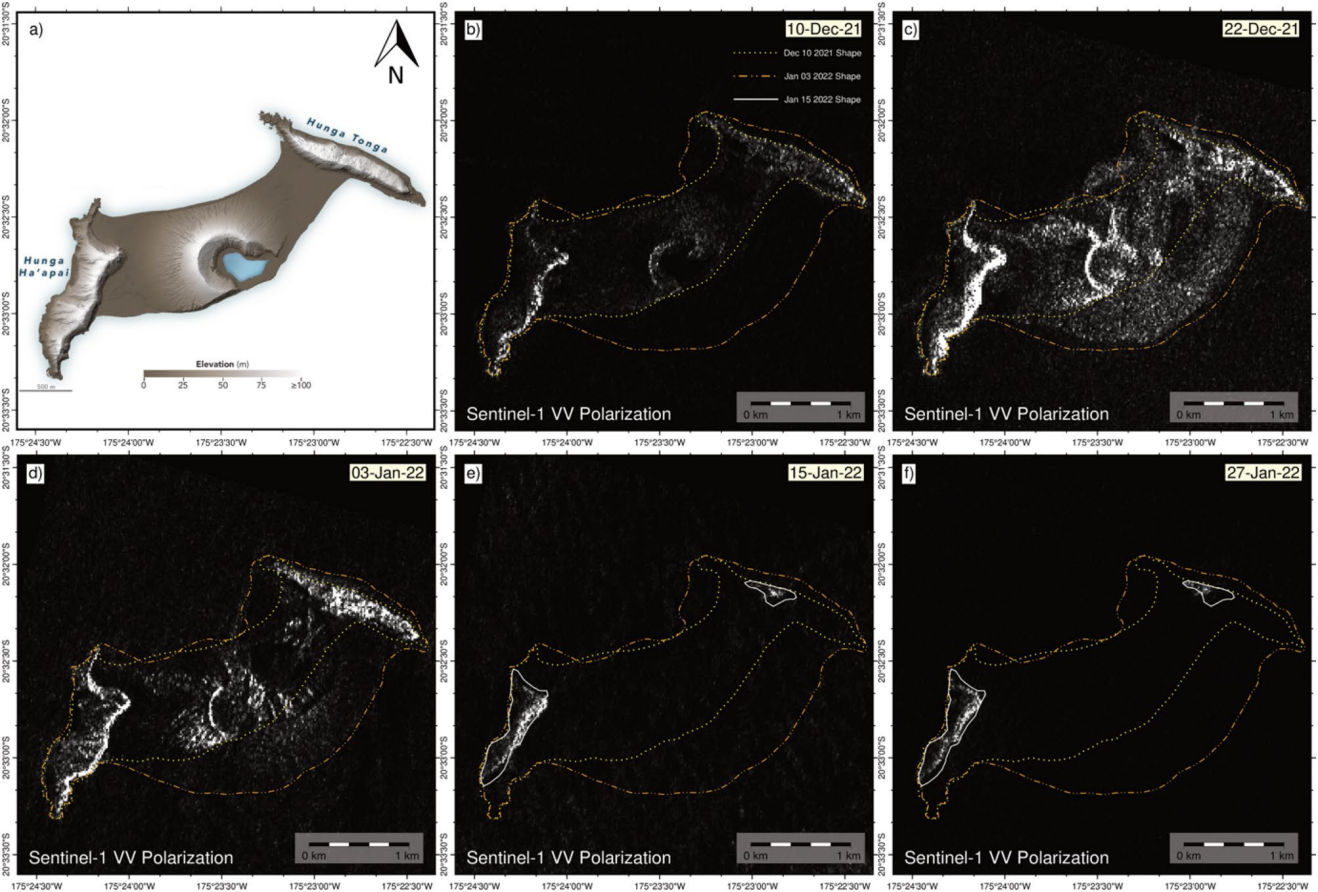
Morphological changes at the Honga Tonga-Hunga Ha’apai island detected by SAR amplitude images VV polarization. Descending images are used for the generation of the images comparing them with (**a**) Modified Digital Elevation map April 2021 from NASA Earth Observatory^56^. Our processed Sentinel-1 amplitude maps (**b**) on December 10, 2021, (**c**) on December 22, 2021 and (**d**) on January 3, 2022 show the dramatic changes before the explosion. The yellow, orange, and white contours show the changes in the morphology of the island. Figure (**e,f**) show the shape change of the island after the January 15. 2022 volcanic eruption. This Figure was created using the Generic Mapping Tools (GMT) version 6.0.0 (https://www.generic-mapping-tools.org).

The stretched area of the Tonga island appeared after the 2014–2015 chain of the volcanic eruption^53^. The new island formed between two older Tonga islands (Hunga Tonga to the NE and Hunga Ha’apai to the SW; Fig. 3, on the rim of the ~ 5 km diameter submarine Hunga Caldera^54^. High spatial resolution satellite observations by Airbus’ Pléiades demonstrated that the resulting island had a total new land area of ~ 1.74 km^2^ (1.94 km^2^ including the interior crater lake) and relief of ~ 120 m (Fig. 3A)^53^. Typical ash-dominated eruptions rarely produce island landscape systems that survive for more than several months^55^. So, as in the 2014–2015 explosion Surtseyan eruption^27^, the ash blow-out dominated most of the new island washed away within a few months due to intense marine abrasion. After the 2022 eruption, we detected ground surface changes in the center of the island observed by SAR amplitude VV polarization images (Fig. 3b,c). This suggests that by December 22, 2021, an important reshaping of the island had started, which is also detected in the Sentinel-1 in the amplitude images on January 3, 2022, (Fig. 3d). The transition of the reshaping in the Tonga island is shown by the images from December 10, 2021 (Fig. 3b) to January 03, 2022 (Fig. 3d), where an increase in the island size and the beginning of volcanic activity is observed. We estimate the island area with morphological map after the April 2021 (Fig. 3a) till the January 03, 2022 (Fig. 3b). The result shows that the area of the island is increased to ~ 1.88 km^2^. However, the images immediately after the HTHH volcanic eruption on January 15, 2022 (Fig. 3e) and January 27, 2022 (Fig. 3f) reveal a very quick disappearance/submergence of the island. However, the northeast and southwest sides of the obliterated island remain above sea level (Fig. 3e,f).

## Discussion

On Saturday, January 15, 2022, the HTHH volcanic eruption reached its maximum strength with the explosion that took place at 4:15 UTC time. Preliminary data show that the event was the largest volcanic eruption in the twenty-first century and the largest since the 1991 Mount Pinatubo eruption^57^. The United States Geological Survey (USGS) reported an earthquake of M_w_ 5.8, 68 km NNW of Nuku’alofa, Tonga, 20.546° S 175.390° W at 4:14:45 UTC. However, it did not report any moment tensor or focal mechanism solution. A tsunami followed the explosion of the volcano, where an air water coupling mechanism was responsible for triggering faster speed, longer duration, and larger amplitude waves recorded at distant locations, compared to the expectation from seismic sources^58^. According to the more frequent seismic-induced tsunamis, the energy and amplitude of tsunami waves depend on the initial sea-surface perturbation produced by the sea-bed deformation along the plate interface rupture, which defines the amount of energy transfer from the earthquake to the water column^59^. After the initial energy transfer, the tsunami propagates as a long water wave, whose speed (and thus refraction) is mainly controlled by the bathymetry^60^. In the case of the HTHH tsunami, sea surface records show faster, long-lasting, and unexpectedly large tsunami waves, which may have resulted from the interaction of acoustic gravity waves (caused by the explosion) with sea surface water internal gravity waves (propagating as a tsunami from the source area) that exchanged energy through resonant mechanisms^58,61–68^ and atmospheric Lamb wave moving away from the volcano in concentric circles at approximately ~ 350 m/s^37^.

### Internal gravity waves and tsunami signature

Generally, in the tsunamigenic earthquake, the ionospheric TEC perturbations are associated with low frequency acoustic gravity waves^43^ (velocity 800–1500 m/s), which are directly generated by the surface uplift, Rayleigh waves^10,21^ (velocity 3300–4000 m/s), S-waves^69^ (velocity 3000–4000 m/s), small-amplitude P-waves^70,59^ (velocity 5000–7000 m/s), and internal gravity waves initiated by tsunamis^43,60^ (velocity 200–300 m/s). However, in the HTHH volcanic eruption case the scenario is different. We detected two acoustic gravity waves with a speed of ~ 450 and ~ 750 m/s. It suggests that the surface deformation was instantaneous in the seconds to minutes like an earthquake. The deformation was in the form of chain of volcanic eruption. We also observed the internal gravity wave signatures in the residual VTEC. Its intensity shored a directional spread. In the Fig. 2B, the wavelet analysis suggests that internal gravity waves reach at all the GNSS sites (see Fig. S1), and first at the closest ftna GNSS site located in the NNW direction of HTHH volcanic eruption (Fig. 2B). Its period of ~ 60 to 80 min from 4:30 UTC to 8:00 UTC, but the intensity is relatively lower than the internal gravity wave signal at wark GNSS site located ~ 2000 km in the south direction. The internal gravity wave signature at the wark GNSS site is very intense with a period between ~ 40 to 60 min during 5:00 UTC to 7:00 UTC. In the east, GNSS site nrmd shows a weak internal gravity wave signal of period ~ 40 to 60 min and 80–100 min from 5:30 UTC to 8:00 UTC. The higher periodic wave 80–100 min is much weaker than the lower periodic wave. In the west direction GNSS site thti located around ~ 2800 km exhibits a very low intensity of internal gravity wave of the period 20 min to 40 min from 6:00 UTC to 10:00 UTC. Overall, the analysis suggests that internal gravity waves were strongest in the south and west direction and have significantly correlated with the tsunami propagation direction.

**Figure 4:**
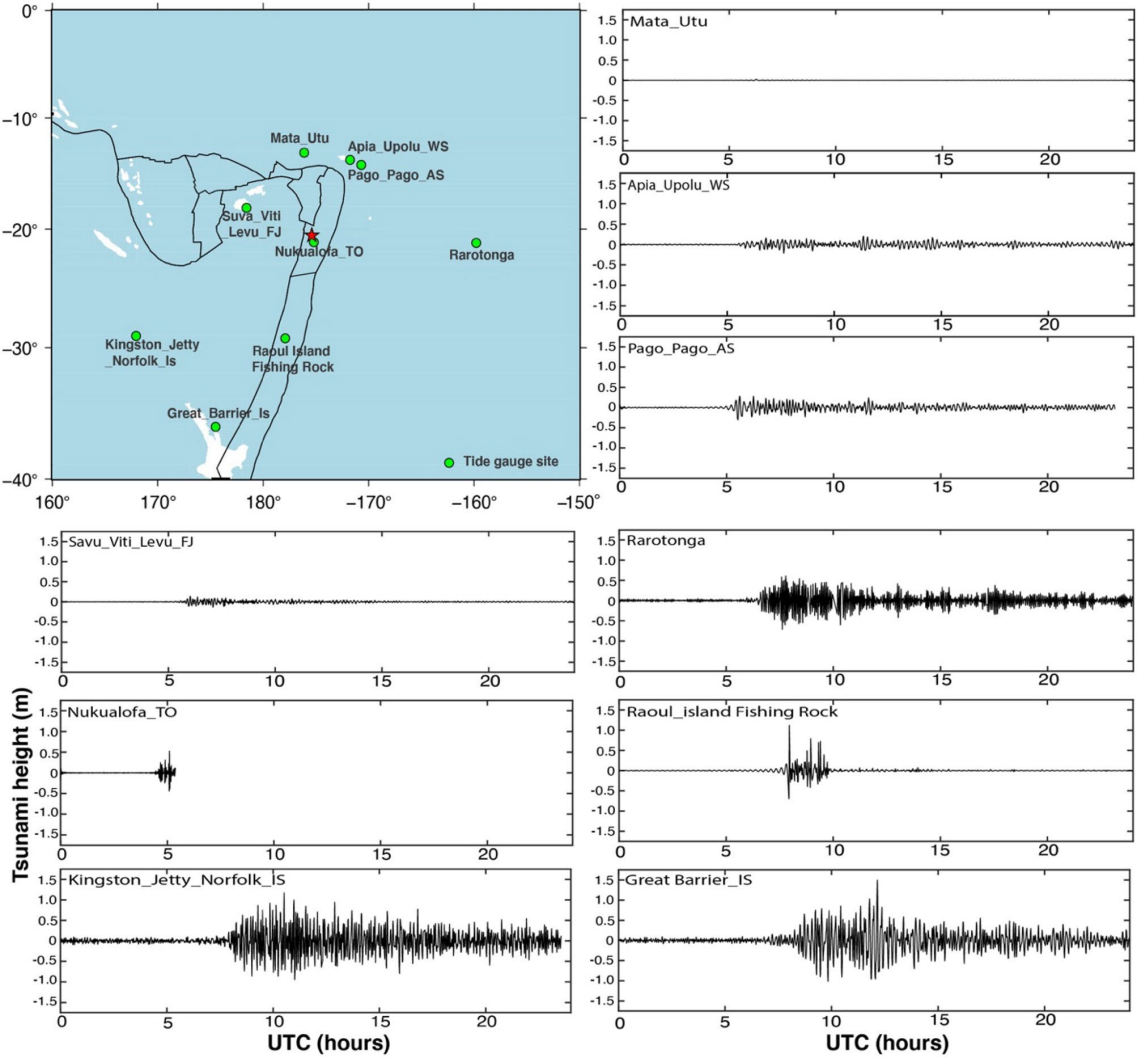
Tide-gauge locations in the Tonga region (left top panel). The tsunami waveform for the HTHH volcanic eruption is shown in the remaining panel. The data were filtered using a zero-phase digital high-pass filter to eliminate signals over a 180 min period. The star represents the HTHH volcanic eruption 2022. This Figure was created using the Generic Mapping Tools (GMT) version 6.0.0 (https://www.generic-mapping-tools.org).

The tsunamis were observed all around the HTHH volcanic eruption zone, but intensities varied in different directions. The closest tide gauge site located around 70 km Nukualofa_TO observe a tsunami height around 0.55 m at 5:00 UTC (Fig. 4), but this tidal gauge stopped recording shortly after. The second nearby tide gauge site Suva_Viti_Levu_FJ is observed with a maximum tsunami height of 0.14 m in the WWN of HTHH volcanic eruption vent. In NNE, the two tide gauge sites, Apia_Upolu_WSand Pago_Pago_AS located very close to each other recorded tsunami height 0.20 m and 0.29 m respectively. At the same time in the NNW, tide gauge site Mata_Utu detected a very weak signal of the tsunami. Further in the south and SSW, there are three tide gauge sites recorded more than 1.0 m tsunami height. Likewise, in the eastern side, the Rarotonga_CK tide gauge site observed a maximum tsunami height of 0.60 m. It may be noted that the tsunami signal amplitude is varying in complex fashion in the different directions^68,71^ with a more dominancy towards in the south direction. It is also noticed that the tsunami reached all of the pacific rim regions^58,72^. The internal gravity wave induced TEC data and tsunami observations at tide gauge site Great barrier_Is located in the New Zealand region around 1950 km, shows that the internal gravity wave was more dominant from 5:00 UTC to 7:00 UTC. However, the tsunami wave was more dominant from 9:00 UTC to 11:00 UTC. It also infers that the amplitude of internal gravity wave from the GNSS derived TEC followed by the tsunami propagation same as in the case of tsunamigenic earthquake^10^. This suggests that the internal gravity wave reached the sites 4 h prior to tsunami waves. The Great barrier_Is tide gauge sensor 1950 km received another train of tsunami waves later after 11:00 UTC. It suggests that the tale of tsunami waves reaches at tide gauge sites with a superimpose of tsunamis waves^68^. However, the tsunami passed through Raoul_Island Fishing Rock tide gauge site located 1000 km far from HTHH volcanic eruption zone and detected a tsunami height of ~ 1.2 m. The amplitude of tsunami simulated by Pakoksung et al.^73^ based on a submarine explosion scenario constrain with the geometry and magnitude of the explosion energy source based on analyses of pre‐ and post‐event satellite images depicts a good match with our tide gauge observations.

### Acoustic gravity wave as the signature of displaced central part of Tonga Island

In Fig. 2C, wavelet analysis demonstrates that all the GNSS sites recorded the acoustic gravity wave signals. These acoustic gravity signals were having periodicities from 2 to 6 min. The acoustic gravity signals of all the GNSS sites are shown in the supplementary information in Fig. S2. We provided the acoustic gravity wave signal signatures of the PRN10 for nrmd, ftna, thti and wark in Fig. 2C. The acoustic gravity wave signals are very prominent from 5:45 UTC to 9:00 UTC at GNSS site ftna. The intensity of acoustic gravity wave signal at ftna GNSS site increases gradually and intensifies from ~ 7:15 UTC and terminates at ~ 9:00 UTC. After 9:00 UTC, the signal of the acoustic gravity wave terminates. This can be observed with all the PRN’s provided in the supplementary information (Fig. S2).

The acoustic gravity waves associated with the surface deformation observed in the subduction and collision zones earthquakes are well studied in Pisagua 2014 M_w_ 8.1 by Reddy et al.^44^ and Shrivastava et al.^10^, Illapel 2015 M_w_ 8.3 by Reddy et al.^46^ and Shrivastava et al.^10^ and Nepal 2015 M_w_ 7.9 by Reddy and Seemala^45^. The moment rate function of the Pisagua, Illapel and Nepal earthquakes provided that seismic moments are released within ~ 2 min, ~ 3 min and ~ 1 min respectively. Consequently, the maximum uplift of 1.20 m and maximum subsidence of 0.25 m estimated in the Pisagua, maximum uplift of 2.00 m and maximum subsidence of 0.2 m in the Illapel and maximum uplift of 1.0 m and subsidence of 0.8 m in the Nepal earthquake. The wavelet analysis of the TEC perturbations in the earthquake studied by (Shrivastava et al.^10^, Occhipinti et al.^43^, Reddy et al.^44^, Reddy and Seemala^45^, Reddy et al.^46^, and Ravanelli et al.^19^) suggested that the acoustic gravity wave appears after 8 min of the main shocks. The Pisagua and Illapel subduction earthquakes also exhibit the acoustic gravity wave signatures ~ 20 min continuously appeared in the wavelet analysis. Similarly, Reddy and Seemala^45^ identified acoustic gravity waves ~ 20 min in the Nepal earthquake in the collision zone with the surface deformation. Therefore, the acoustic gravity wave signals can be correlated well with the surface deformation signals. Sometimes, in the case of earthquake the propagation of coseismic Ionospheric perturbation, the geomagnetic field and background ionization reorient the phase and amplitude of ionospheric perturbation. It can be non-tectonic forcings that affect ionospheric perturbation identified by Chum et al.^74^ in the 2015 Illapel earthquake. The shape and amplitude of ionospheric perturbation may vary due to the non-linear behavior of quasi-vertically propagating infrasound waves^74^.

We do not have the direct measurement of surface deformation in the Tonga volcanic region. Therefore, we propose the pattern of surface deformation utilizing the observation of acoustic gravity wave. The acoustic gravity wave signals at GNSS sites of ftna of PRN’s 10, 23, 24 and 32 recorded in different times. During the volcanic eruption PRN 23 and 24 were available at high elevation angles in the range of 60°–90°. These PRN’s received acoustic gravity wave signals ~ 4:25 UTC time just after the 10 min of the eruption. On the other hand, PRN 10 and 32 were at low elevation angles. These PRN’s recorded the acoustic gravity waves signals from 6:00 UTC time until 9:30 UTC time. It means the deformation started along with volcanic eruption and it continued untill 9:30 UTC time. The acoustic gravity signals of these PRN’s are demonstrating a desecrate fluctuation from the GNSS TEC perturbation, indicating that the volcanic explosion was an intermittent event similar pattern also identified by Astafyeva et al.^32^. This acoustic gravity wave signal suggests a subsidence associated with the central part of the Tonga island, which appeared during the 2014–2015 volcanic eruption.

### Mechanism of atmospheric Lamb wave

In general, there are two main waves as acoustic gravity wave and internal gravity wave observed in the residual VTEC of ionosphere. But during the volcanic eruption, atmospheric Lamb wave also observed. The atmospheric Lamb wave propagates with speed around 300–350 m/s in the troposphere. The atmospheric Lamb wave was first observed in the Krakatoa eruption 1883^25,26^. Similar pattern of the Lamb wave was detected during the St. Helens eruption 1980^75^. Therefore, atmospheric Lamb wave propagated globally with the speed of 300–350 m/s is consistent with previous results. This wave works as main transporter for eruption energy in the ionosphere and upper atmosphere. Hence, we also suggest that the volcanic eruption energy propagate upward with circular concentric wave and perturbed the ionosphere. The expansion of the circular pattern of atmospheric wave exponentially increased with the height due to decrease plasma density.

## Concluding remarks

We study the GNSS-derived ionospheric TEC perturbation, SAR-derived volcanic eruption morphology, tide gauge-derived tsunami propagation associated with January 15, 2022 HTHH volcanic eruption event within the radius of ~ 5000 km radius. The internal gravity wave has confirmed the dominant tsunami propagation direction in the south and west direction. It shows that HTHH volcanic eruption explosion uplifted the water column along with the huge plume and ash. We also detected an acoustic gravity wave which is more dominant in the north direction from 5:45 UTC to 8.45 UTC. The acoustic gravity wave signature appears in the TEC around 5:25 UTC after 10 min of the volcanic eruption. After 7:15 UTC the intensity of the acoustic gravity wave was very prominent. Sentinel-1 SAR data demonstrates that 5 h after the volcanic eruption the central part of the HTHH island landscape disappeared with the biggest atmospheric explosion. It postulates that the acoustic gravity wave signals observed in TEC are associated with the subsidence in the central part of the Tonga Island. Further, Lamb wave, observed in the data, support existing theories that Lamb wave leak in to the thermospheric altitudes through the atmospheric resonance channels at acoustic and gravity wave frequencies, carrying strong disturbances at high altitudes^65^.

## Methods

### TEC derived from GNSS data

We have used the TEC measurements derived from GNSS observations data to study ionospheric TEC changes associated with the HTHH volcanic eruption. TEC is an integrated measure of electron density along the satellite-receiver line of sight. Slant TEC (line of sight TEC) estimation from GNSS observation data are performed using the formula,

1 $${\text{STEC}} = \frac{1}{40.3}\left( {\frac{{{\text{f}}_{1}^{2} {\text{f}}_{2}^{2} }}{{{\text{f}}_{1}^{2} - {\text{f}}_{2}^{2} }}} \right)\left( {{\text{L}}_{1} \uplambda_{1} - {\text{L}}_{2} \uplambda_{2} } \right),$$

where and *L*_1_ and *L*_2_ are the carrier phase measurements, *f*_1_ = 1575.42 MHz and *f*_2_ = 1227.60 MHz are the carrier frequencies and λ_1_ and λ_2_ are the corresponding wavelengths. The STEC values are converted to vertical TEC (VTEC) by applying the following mapping function^76^.

2 $${\text{M}} = \left[ {1 - \left( {\frac{{\cos ({\text{E}})}}{{1 + {\text{h}}/{\text{R}}_{{\text{E}}} }}} \right)^{2} } \right]^{1/2} ,$$

3 $${\text{VTEC}} = {\text{STEC}} \times {\text{M}},$$

where h is the height of ionospheric piercing point (IPP), E is the elevation angle of the satellite, and R_E_ is the radius of Earth. While estimating the TEC values, we have considered relatively lower elevation satellites by keeping an elevation mask of 10° in absence of high mountains and topographic morphology in the Tonga region. In the present analysis, we have detrended the TEC variations using a polynomial of order seven. To investigate the ionospheric electron density perturbations in the near field of HTHH volcanic eruption.

### SAR data analysis for morphological view of point

The Sentinel1-A TOPS images from descending orbit, acquired from Alaska Satellite Facility (ASF) using Vertex ASF’s data search portal (https://vertex.daac.asf.alaska.edu/) were used to generate SAR amplitude images. We employed beam mode Wide Swath (WS) and level processing Single Look Complex (SLC) data to process the amplitude maps shown in (Fig. 3b–f). We used GMTSAR software^77^ to produce amplitude files generated with the decimation of a factor from 1 in range to 2 in azimuth due to the small target area. The images processed for each amplitude map prior and after the HTHH volcanic eruption on January 03 (Fig. 3c) and January 15, 2022 (Fig. 3d) respectively. The data proved to be an excellent way to follow the morphological changes within the Tonga-Hunga island allowing us to have complementary qualitative information. The InSAR phase was processed with the Two-pass DInSAR technique to try to get the surface deformation before and after the volcanic eruption over the island. It can be explained by the fast reshaping of the island during the eruption.

### Tsunami observations

The sea-level records of the HTHH volcanic eruption 2022 were extracted from the surrounding tide-gauge network (http://www.ioc-sealevelmonitoring.org). We utilized very nearby tide gauge sites data from the HTHH volcanic eruption region to identify the tsunami heights for comparing the gravity waves of ionospheric TEC perturbation. Far-field tide gauge sensor could have sensed the morphology and resonance of the tsunami waves. In our analysis, there were four types of tide gauge sensors available such as Radar (RAD), Aquatrak (AQU), Pressure (PRS) and Primary water level (PWL). For signal filtering, we estimated the theoretical tidal component using a harmonic analysis^78^ and applied a bandpass Butterworth digital filter at 120 min to remove wind waves noise. The residual tsunami height data provided in Fig. 4 along with tide gauge sites on the geographic map. The detail of tide gauge sites and its location provided as Table S2 in Supplementary Information. The raw and filtered data are also provided in the Supplementary Information as Fig. S4.

### Supplementary Information

Supplementary Information.

## Data Availability

The SAR dataset is available at (https://asf.alaska.edu)^41^. The GNSS dataset is available at IGS (https://igs.org). The tide gauge site data is available at (http://www.ioc-sealevelmonitoring.org).

## Code availability

The code generated and/or analyzed during the current study is available from the corresponding author upon reasonable request.
