# Supplementary material for: Tracking tsunami propagation and Island’s collapse after the Hunga Tonga Hunga Ha’apai 2022 volcanic eruption from multi-space observations
Source: Sci Rep. 2023 Nov 17;13:20109. doi: 10.1038/s41598-023-46397-1 (PMC10656493; doi:10.1038/s41598-023-46397-1)
Supplement: Supplementary file 1 — Supplementary Information. [file 41598_2023_46397_MOESM1_ESM.pdf]

Supplementary Information for

Tracking tsunami propagation and Island's collapse after the Hunga Tonga Hunga Ha'apai 2022 volcanic eruption from multi-space observations

Mahesh N Shrivastava[1,2,3*], A. S. Sunil[4], Ajeet K. Maurya[5], Felipe Aguilera[1,2], Simón Orrego[1], P. S. Sunil[4], Rodrigo Cienfuegos[3,6] and Marcos Moreno[3,7]

[1]Department of Geological Sciences, Universidad Católica del Norte, Antofagasta, Chile
[2]Millenium Institute on Volcanic Risk Research - Ckelar Volcanoes, Antofagasta, Chile
[3]Centro de Investigación para la Gestión Integrada del Riesgo de Desastres, Santiago, Chile
[4]Department of Marine Geology and Geophysics, School of Marine Sciences, Cochin University of Science and Technology, Kochi, India
[5]Department of Physics, Babasaheb Bhimrao Ambedkar University, Lucknow, India
[6]Departamento de Ingeniería Hidráulica y Ambiental, Pontificia Universidad Católica de Chile, Chile
[7]Departmento de Ingeniería Estructural y Geotécnica, Pontificia Universidad Católica de Chile

*E-mail: mahesh.shrivastava@ucn.cl

Contents of this file

Tables S1to S2

Figures S1to S4

Table S1: GNSS stations within 5000 km radial distance far from close.

S/N	GPS Station code	Latitude	Longitude	Distance [Km]	Sampling interval (seconds)
1	ftna	-14.30780	-178.1209	752	30
2	nrmd	-22.22833	166.48488	1884	30
3	Wark	-36.43441	174.66278	2012	30
4	Wgtn	-41.32346	174.80589	2486	30
5	owmg	-44.02429	-176.3688	2610	30
6	thti	-17.57706	-149.6064	2725	30
7	mqzg	-43.70274	172.65470	2801	30
8	tidb	-35.39921	148.98000	3839	30
9	gamb	-23.13035	-134.9648	4166	30
10	kokv	20.126	200.335	5016	30

Table S2: Tide gauge sites location and maximum tsunami height far from close.

S/N	Tide Gauge Station code	Sensors	Latitude [degree]	Longitude [degree]	Maximum tsunami height(m)
1	Nukualofa_TO	AQU	-21.136	-175.180	0.55
2	Suva_Viti_Levu_FJ	AQU	-18.134	178.423	0.14
3	Mata_Utu	RAD	-13.170	-176.100	0.03
4	Apia_Upolu_WS	PRS	-13.826	-171.761	0.20
5	Pago_Pago_AS	PWL	-14.276	-170.690	0.29
6	Raoul Island Fishing Rock	PRS	-29.251	-177.903	1.16
7	Raratonga_CK	AQU	-21.200	-159.783	0.60
8	Kingston_Jetty_Norfolk_Is	RAD	-29.059	167.953	1.20
9	Great_Barrier_Is	PRS	-36.189	175.488	1.50

RAD = Radar
AQU = Aquatrak
PRS = Pressure
PWL = Primary Water Level

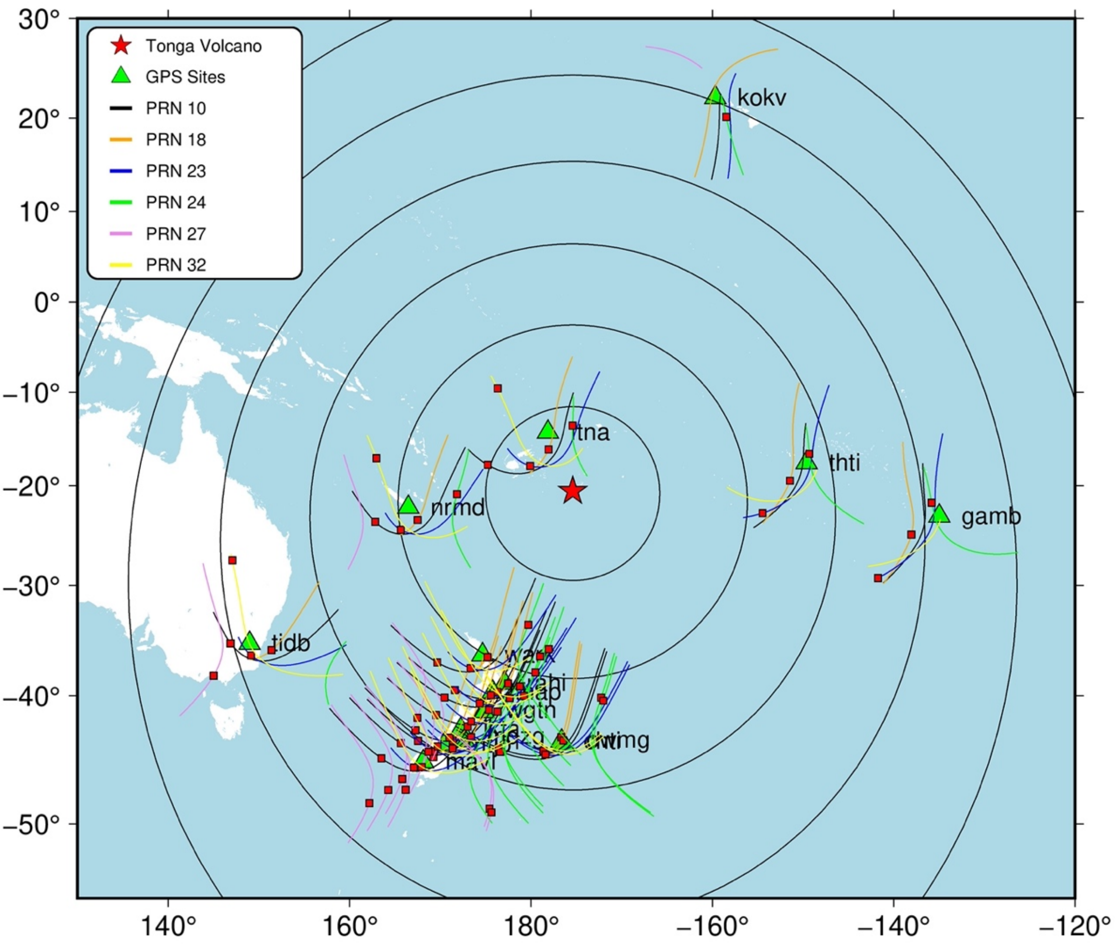

Figure S1: The sub ionospheric point (SIP) tracks of PRN's 10, 18, 23, 24, 27 and 32 and the GNSS sites. The star represents the Hunga Tonga and Hunga Ha'apai volcanic eruption vent. This Figure was created using the Generic Mapping Tools (GMT) version 6.0.0 (https://www.generic-mapping-tools.org).

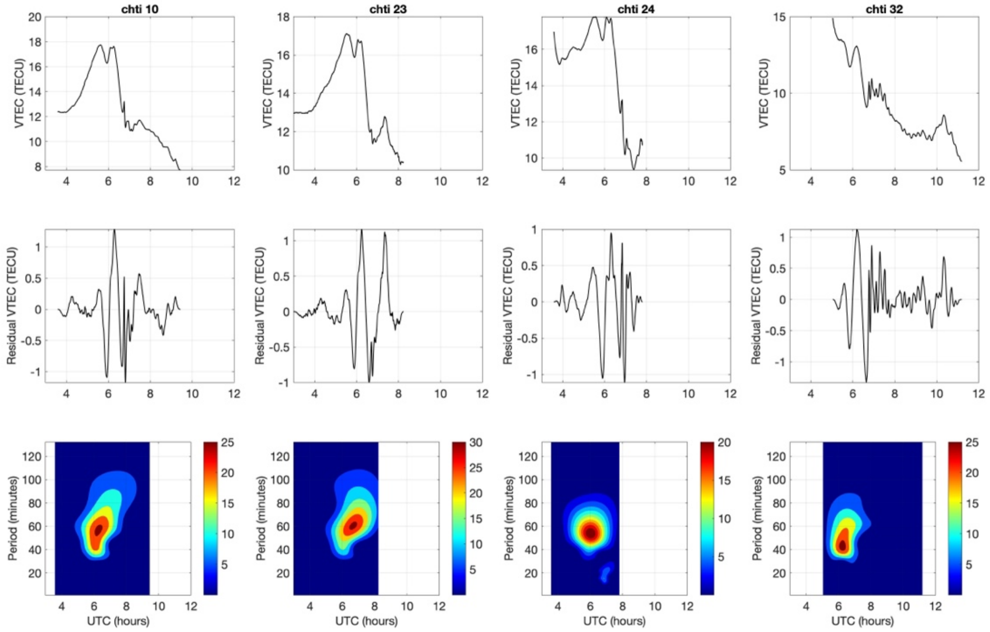

chti 10
chti 23
chti 24
chti 32
VTEC (TECU)
Residual VTEC (TECU)
Period (minutes)
UTC (hours)

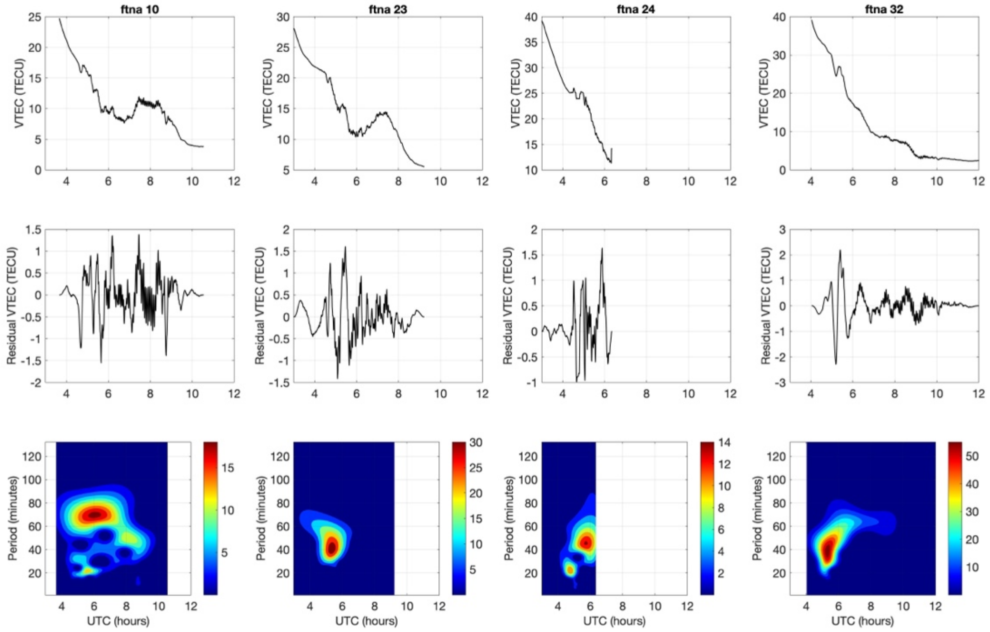

ftna 10
ftna 23
ftna 24
ftna 32
VTEC (TECU)
Residual VTEC (TECU)
Period (minutes)
UTC (hours)

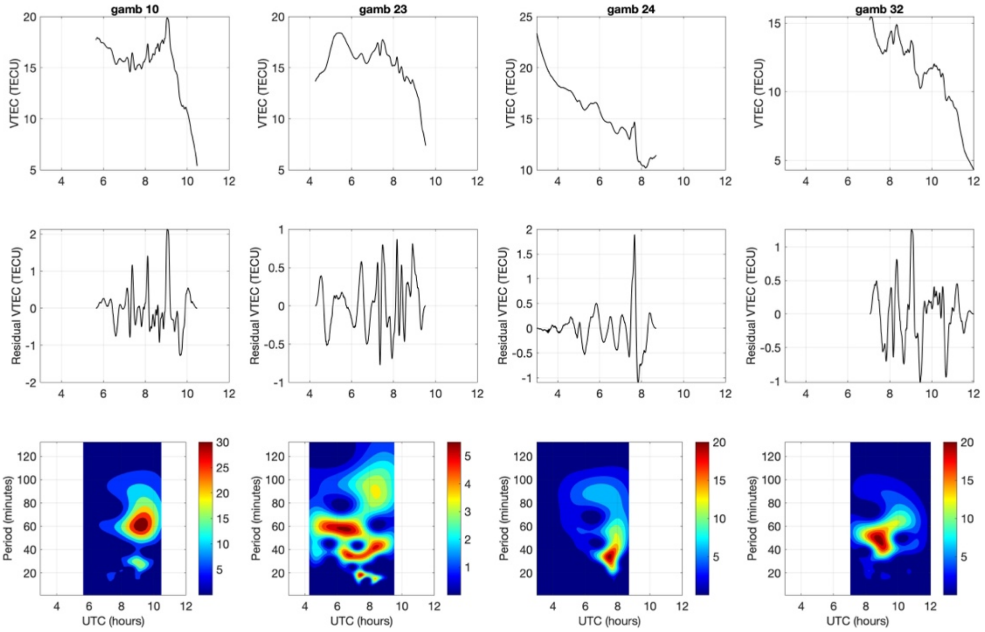

gamb 10
gamb 23
gamb 24
gamb 32
VTEC (TECU)
Residual VTEC (TECU)
Period (minutes)
UTC (hours)

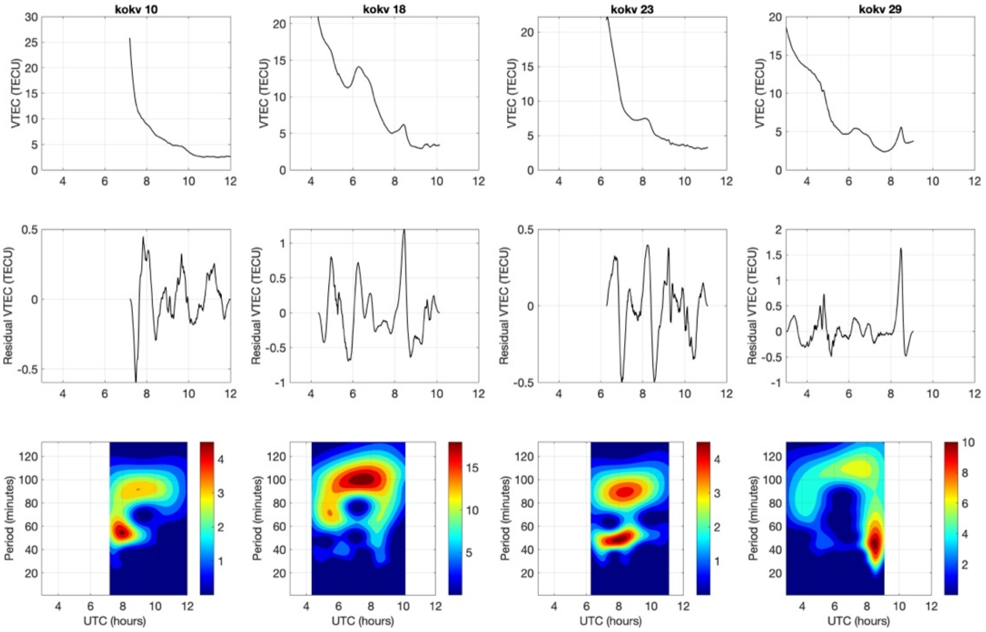

kokv 10
kokv 18
kokv 23
kokv 29
VTEC (TECU)
Residual VTEC (TECU)
Period (minutes)
UTC (hours)

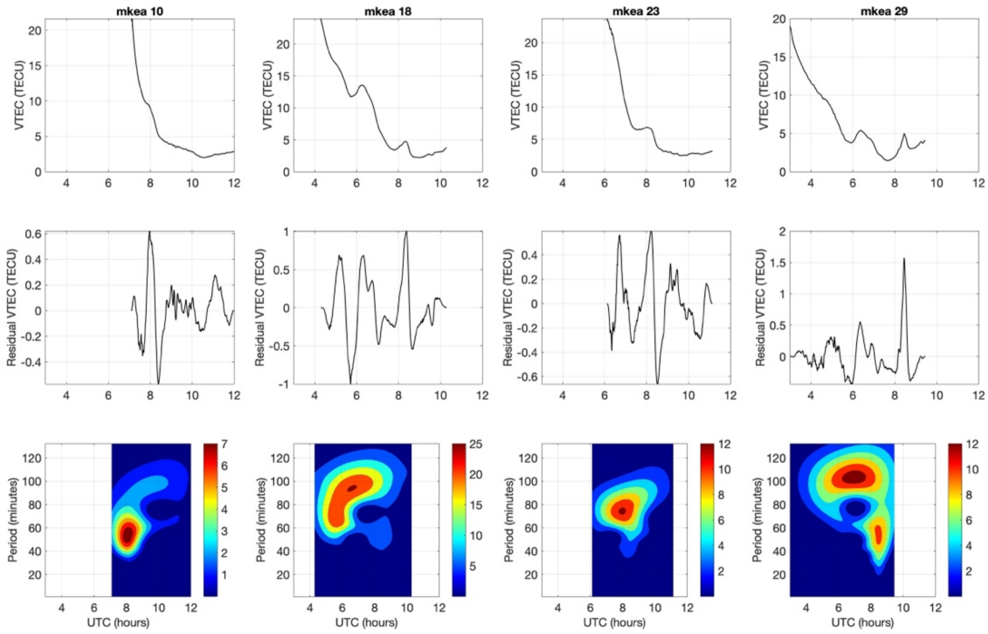

mkea 10
mkea 18
mkea 23
mkea 29
VTEC (TECU)
Residual VTEC (TECU)
Period (minutes)
UTC (hours)

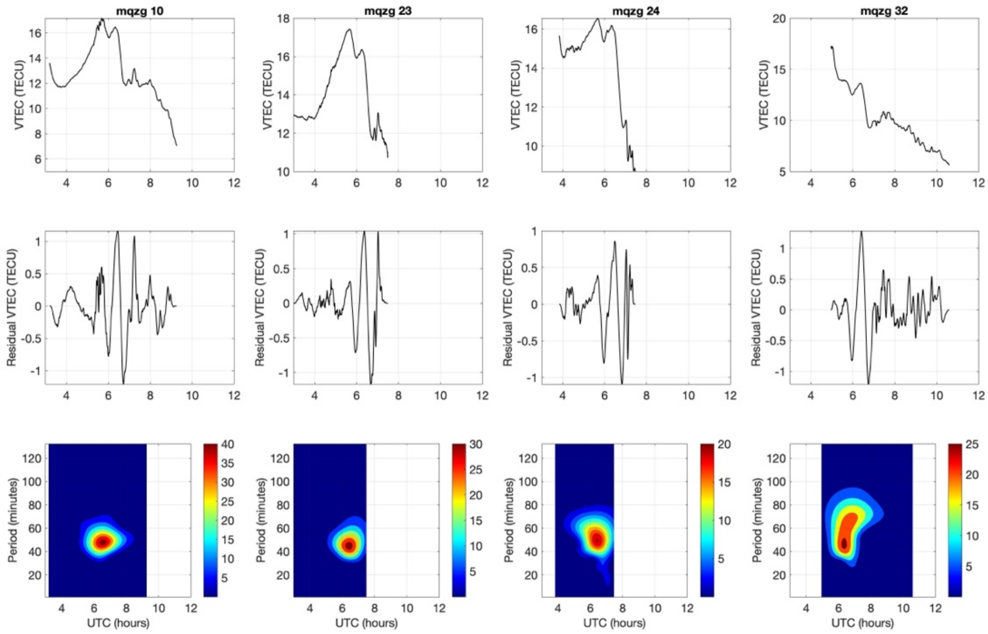

mqzg 10
mqzg 23
mqzg 24
mqzg 32
VTEC (TECU)
Residual VTEC (TECU)
Period (minutes)
UTC (hours)

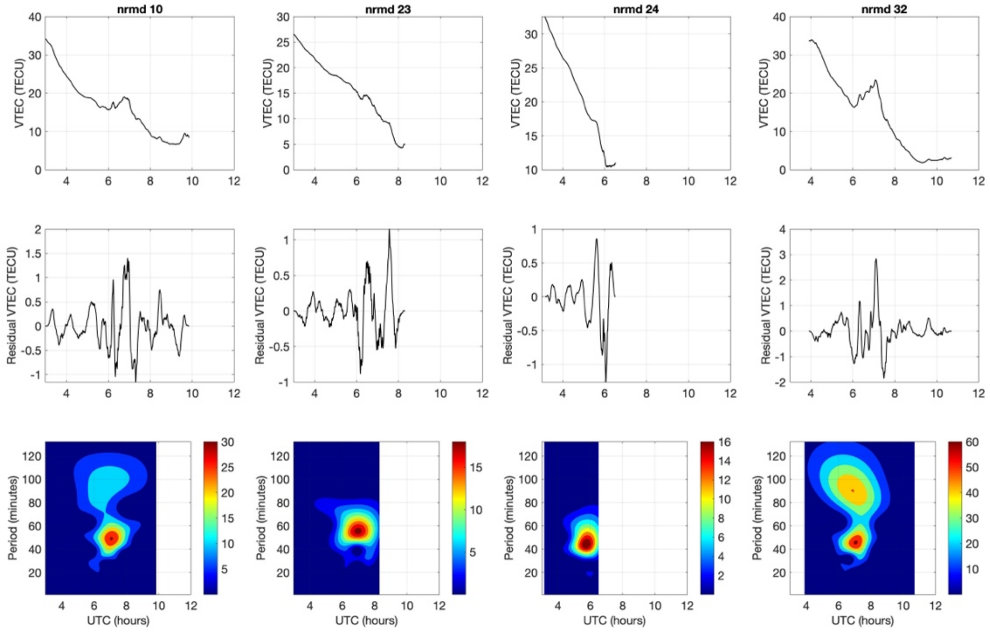
nrmd 10
nrmd 23
nrmd 24
nrmd 32
VTEC (TECU)
Residual VTEC (TECU)
Period (minutes)
UTC (hours)

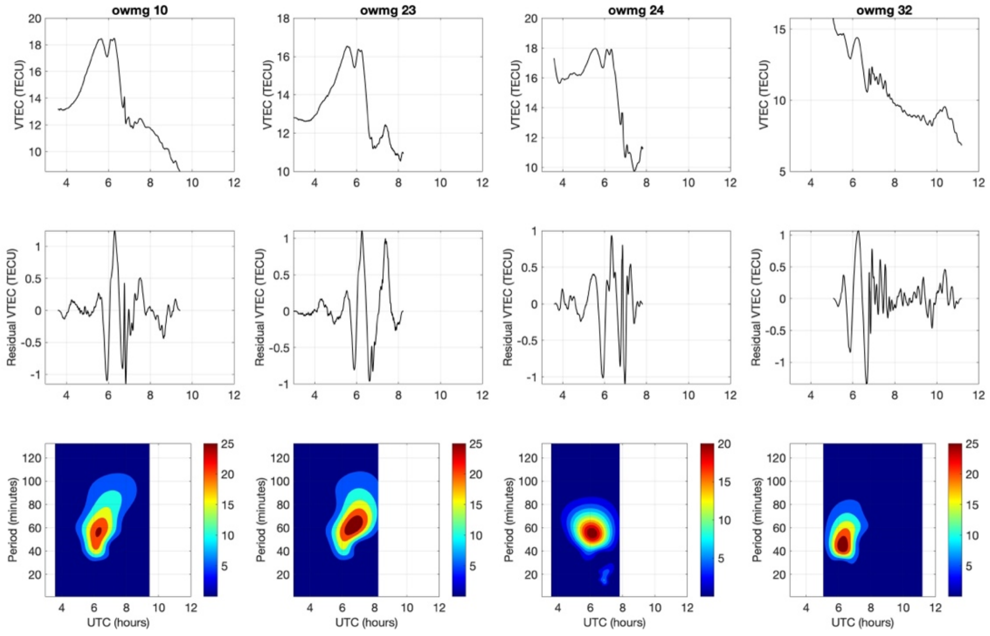
owmg 10
owmg 23
owmg 24
owmg 32
VTEC (TECU)
Residual VTEC (TECU)
Period (minutes)
UTC (hours)

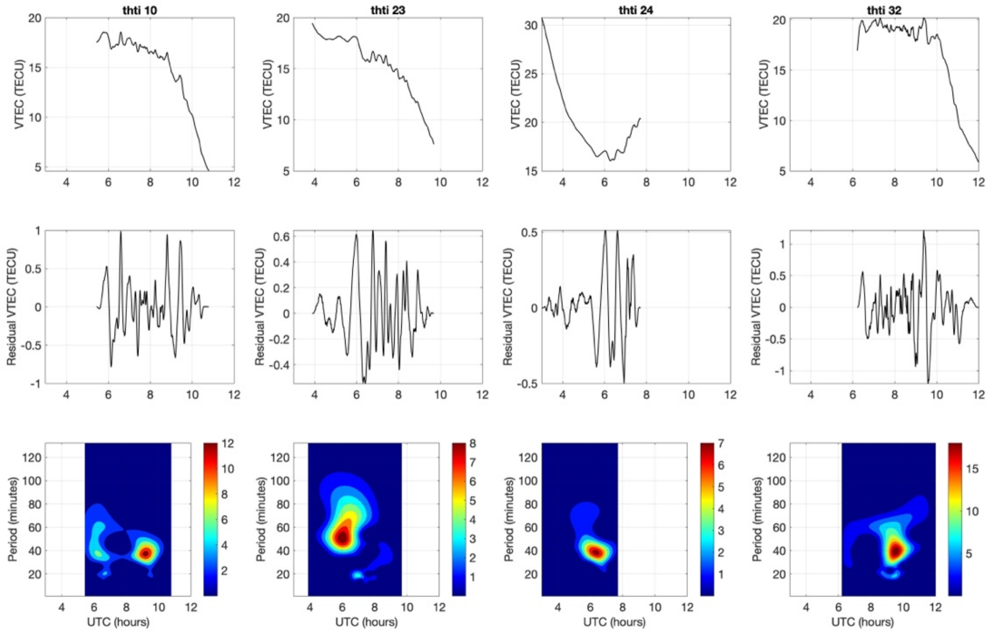

thti 10
thti 23
thti 24
thti 32
VTEC (TECU)
Residual VTEC (TECU)
Period (minutes)
UTC (hours)

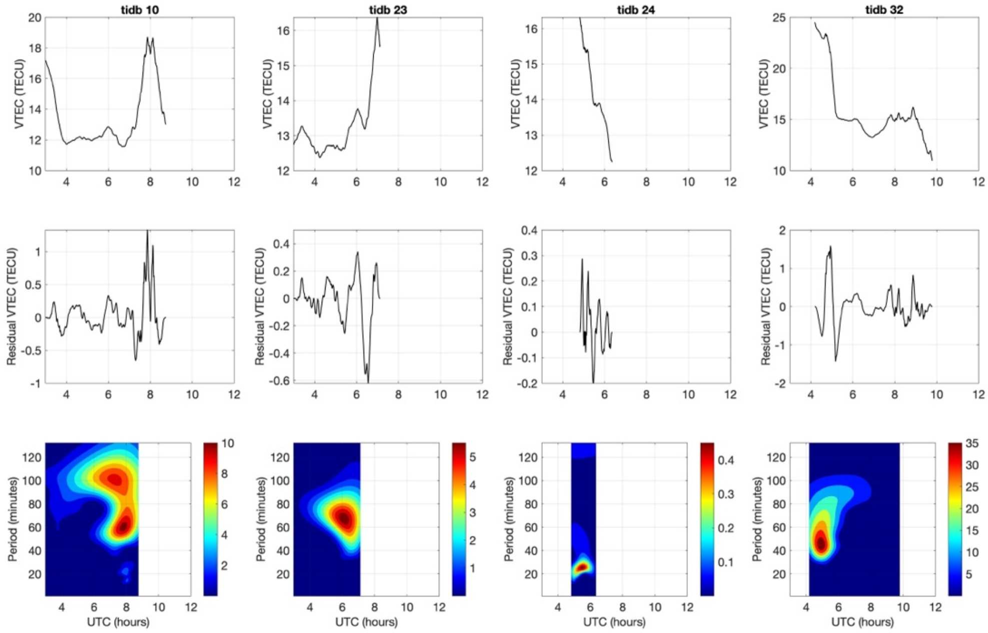

tidb 10
tidb 23
tidb 24
tidb 32
VTEC (TECU)
Residual VTEC (TECU)
Period (minutes)
UTC (hours)

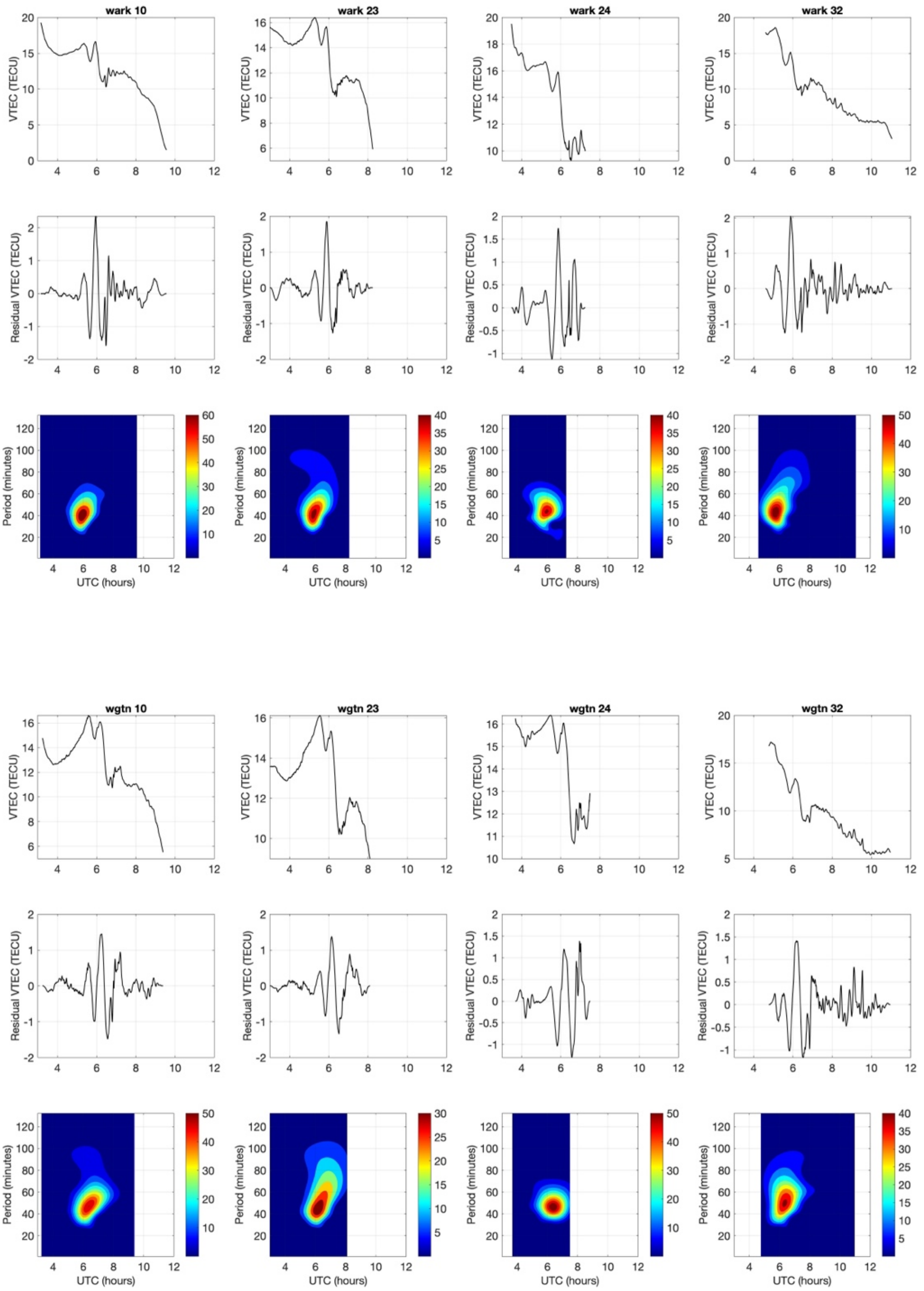

Figure S2: Gravity wave signature in the TEC data at different PRN's at GNSS sites.This Figure was created using the Generic Mapping Tools (GMT) version 6.0.0 (https://www.generic-mapping-tools.org).

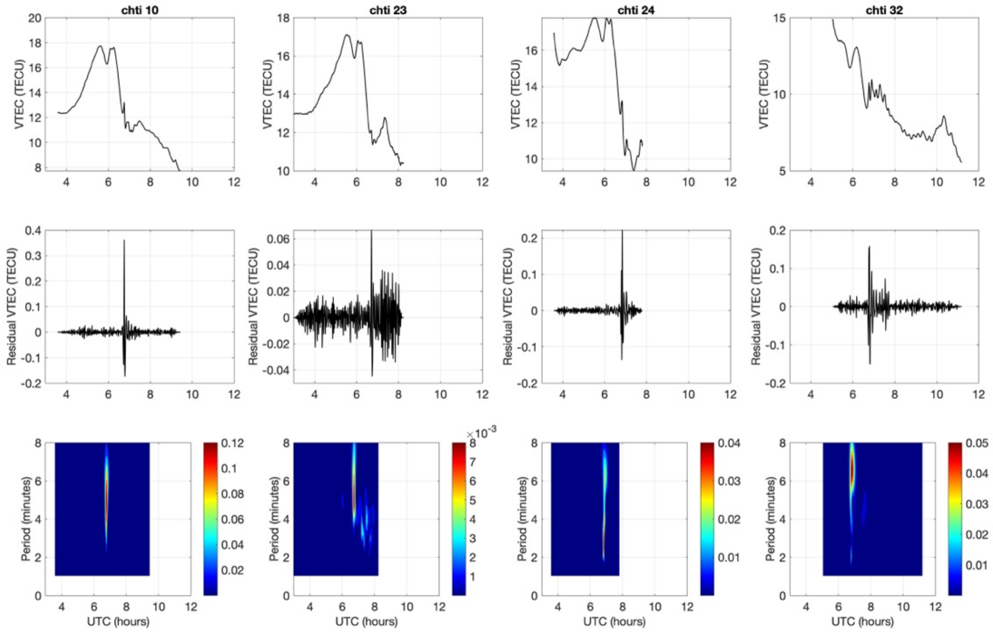
chti 10
chti 23
chti 24
chti 32
VTEC (TECU)
Residual VTEC (TECU)
Period (minutes)
UTC (hours)

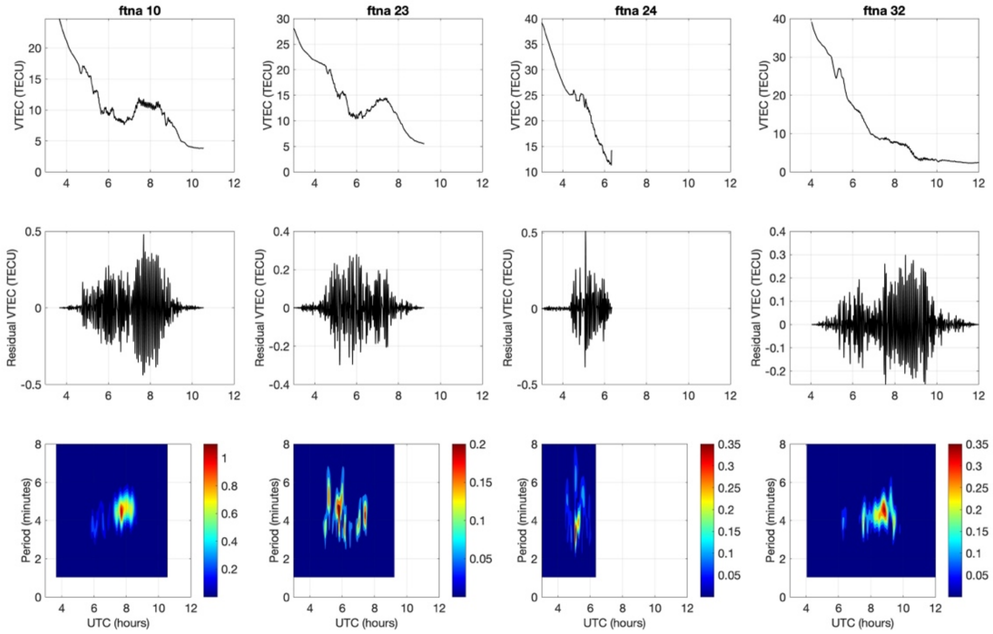
ftna 10
ftna 23
ftna 24
ftna 32
VTEC (TECU)
Residual VTEC (TECU)
Period (minutes)
UTC (hours)

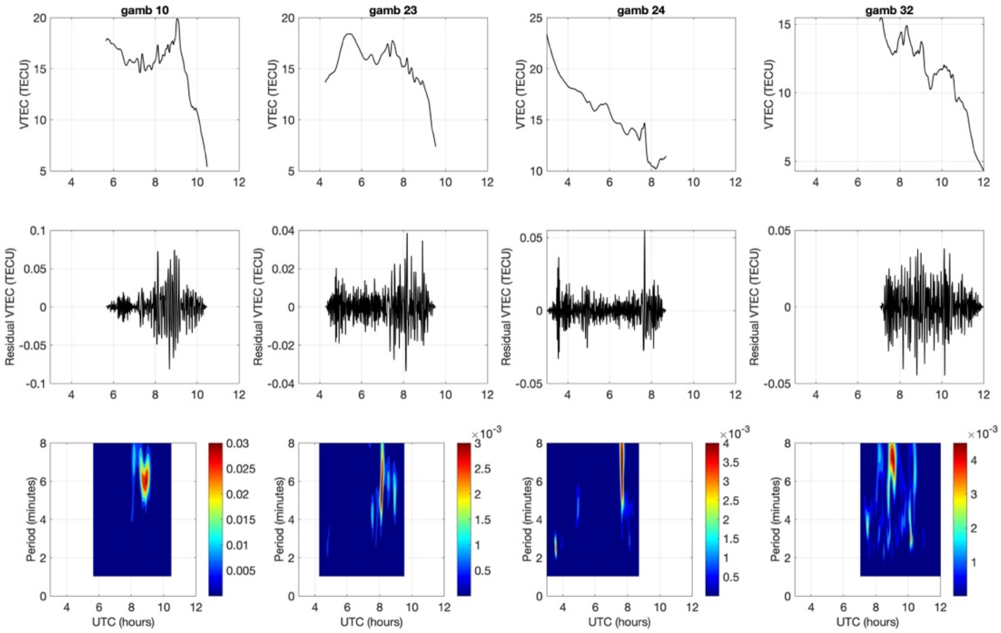
gamb 10
gamb 23
gamb 24
gamb 32
VTEC (TECU)
Residual VTEC (TECU)
Period (minutes)
UTC (hours)

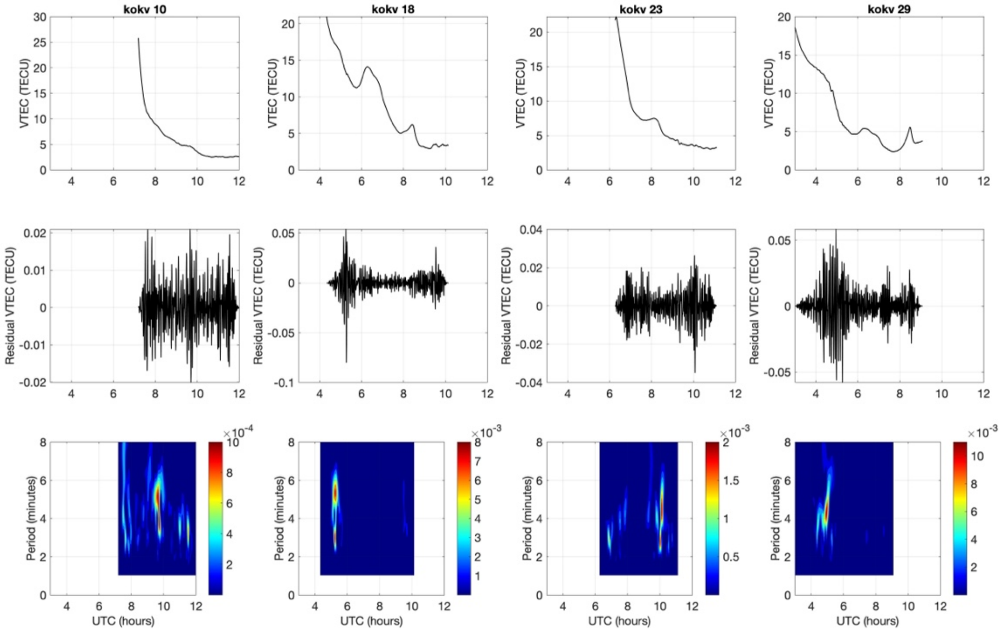
kokv 10
kokv 18
kokv 23
kokv 29
VTEC (TECU)
Residual VTEC (TECU)
Period (minutes)
UTC (hours)

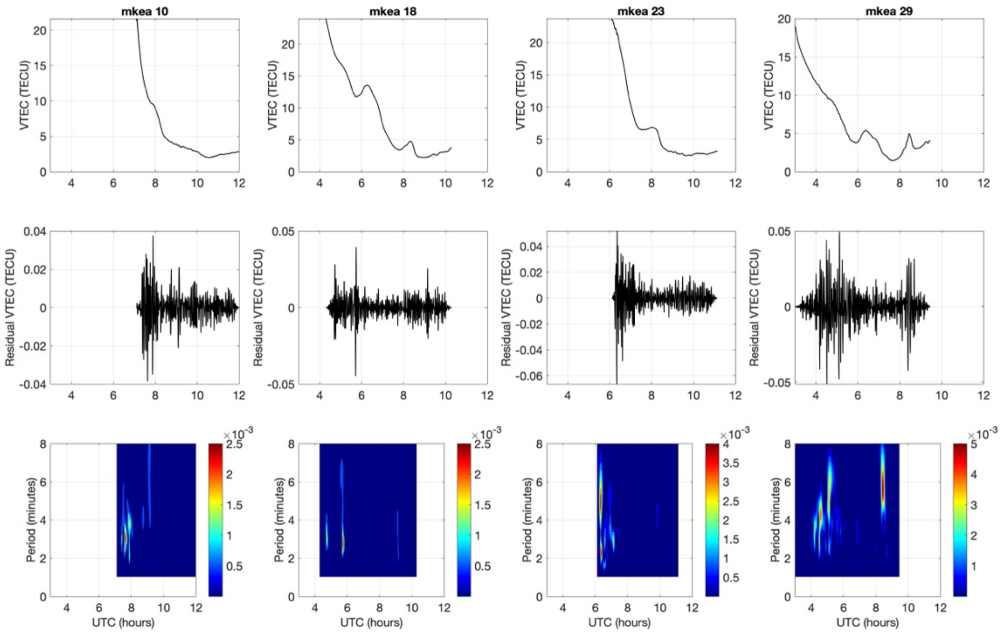
mkea 10
mkea 18
mkea 23
mkea 29
VTEC (TECU)
Residual VTEC (TECU)
Period (minutes)
UTC (hours)
×10^-3

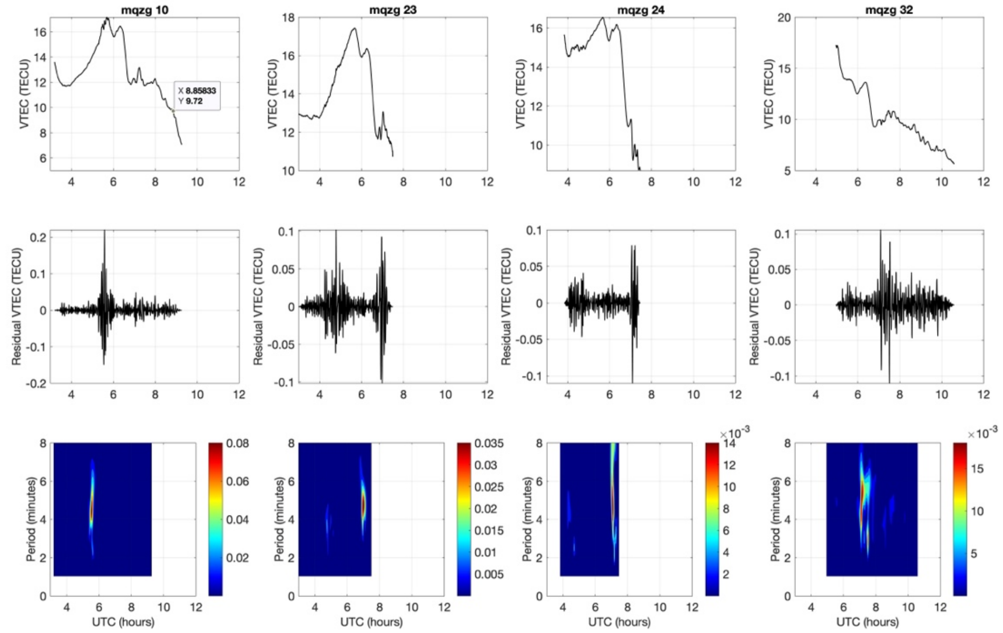
mqzg 10
mqzg 23
mqzg 24
mqzg 32
VTEC (TECU)
X 8.85833
Y 9.72
Residual VTEC (TECU)
Period (minutes)
UTC (hours)
×10^-3

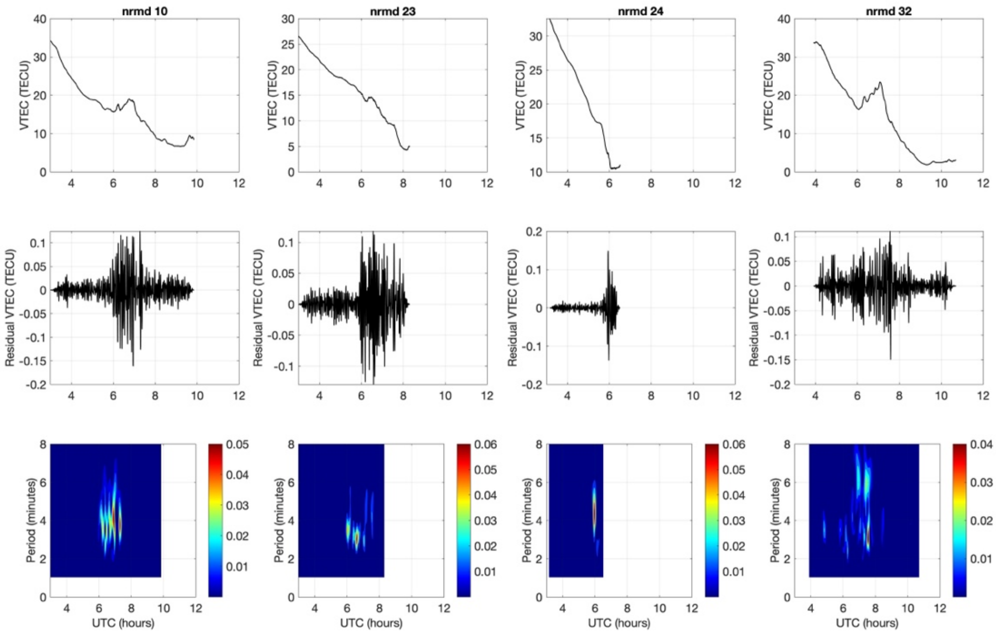
nrmd 10
nrmd 23
nrmd 24
nrmd 32
VTEC (TECU)
Residual VTEC (TECU)
Period (minutes)
UTC (hours)

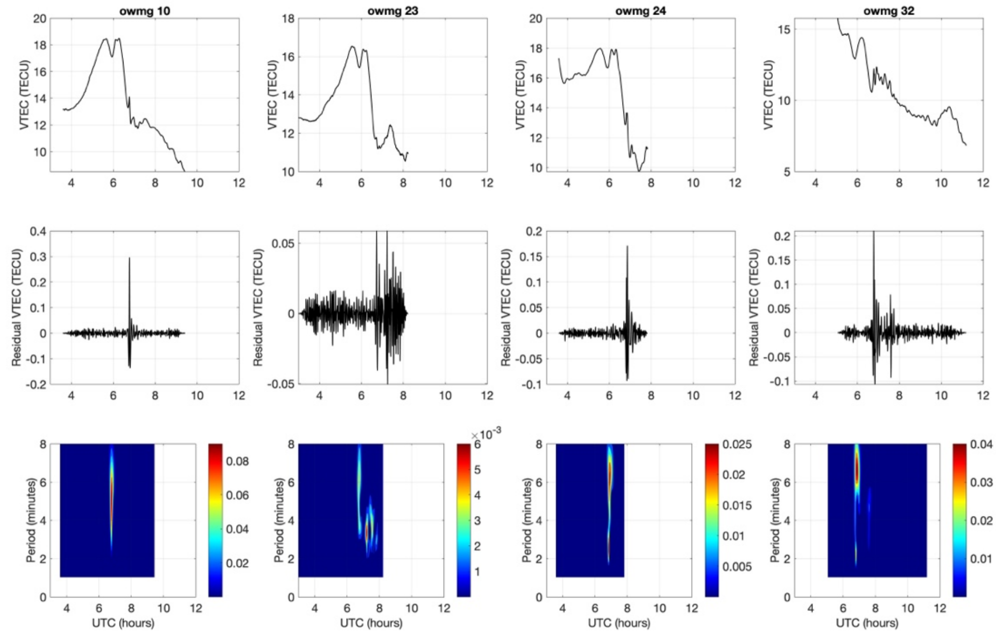
owmg 10
owmg 23
owmg 24
owmg 32
VTEC (TECU)
Residual VTEC (TECU)
Period (minutes)
UTC (hours)

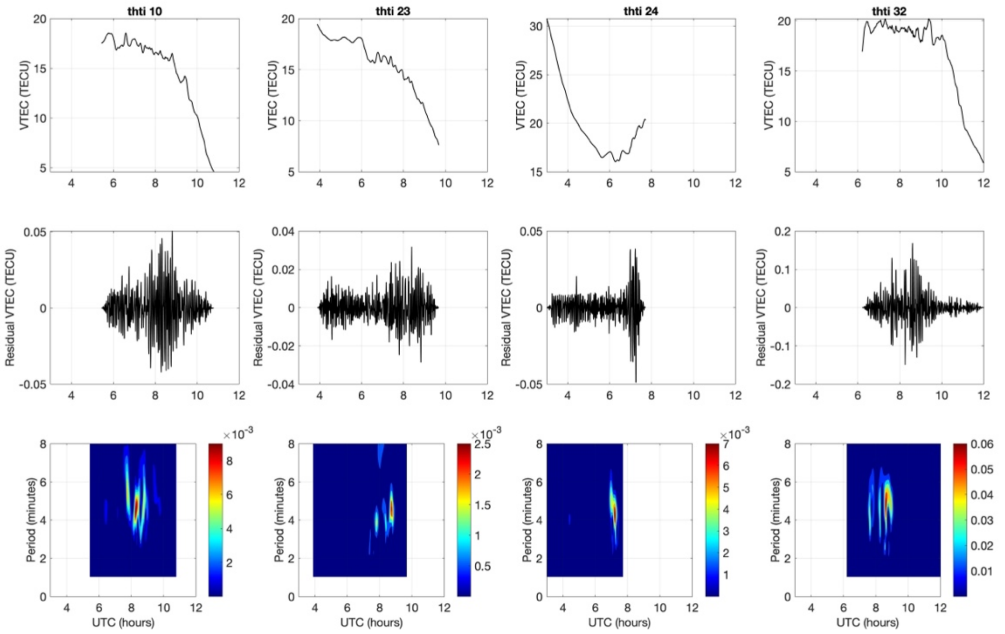
thti 10
thti 23
thti 24
thti 32
VTEC (TECU)
Residual VTEC (TECU)
Period (minutes)
UTC (hours)

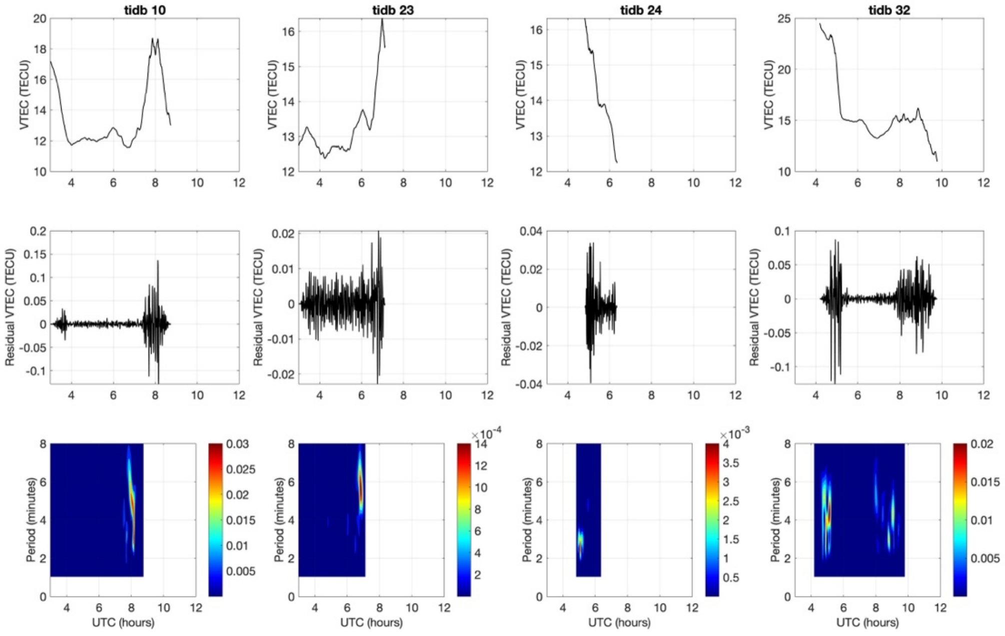
tidb 10
tidb 23
tidb 24
tidb 32
VTEC (TECU)
Residual VTEC (TECU)
Period (minutes)
UTC (hours)

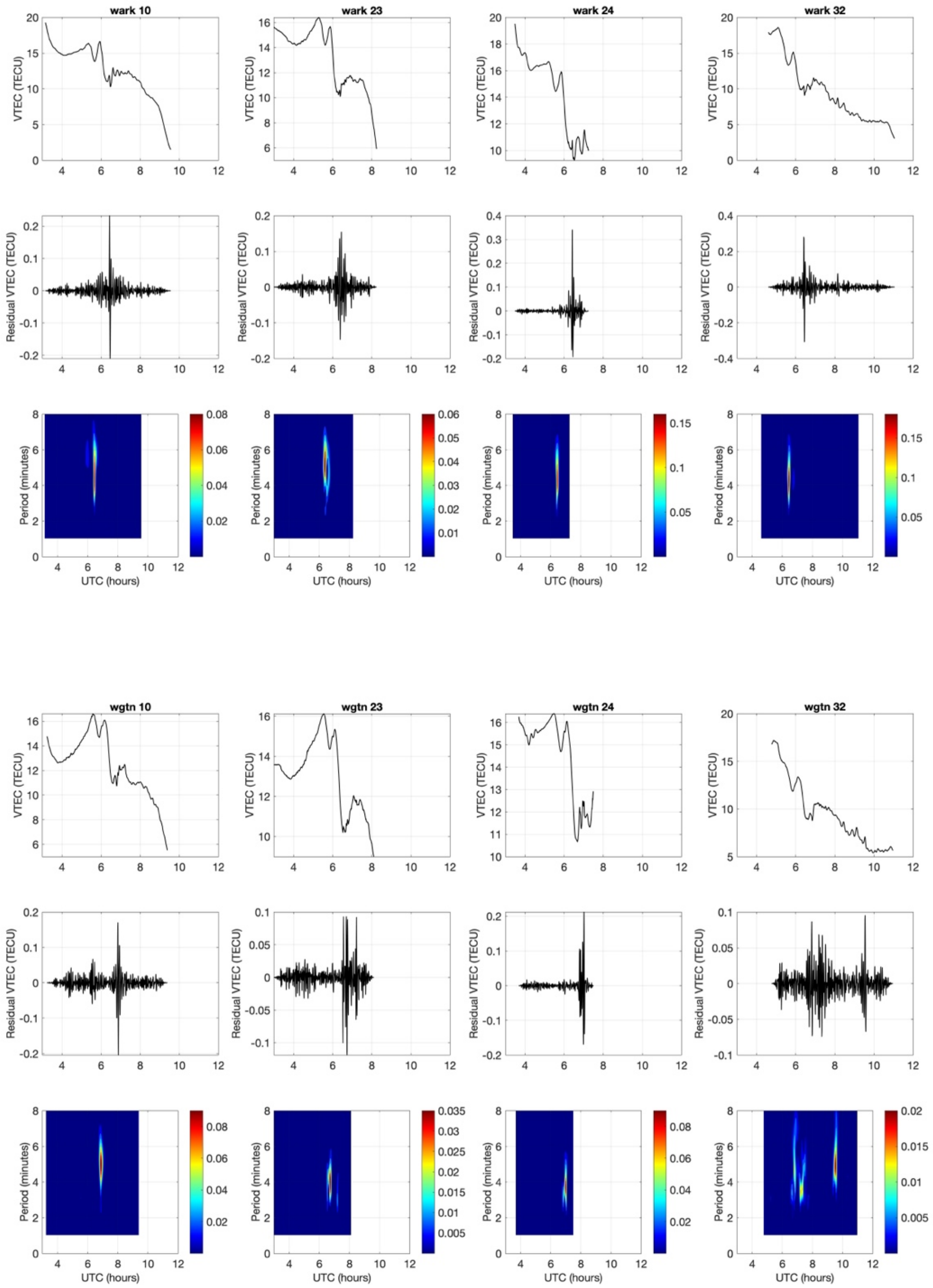

Figure S3: Acoustic wave signature in the TEC data at different PRN's at GNSS sites. This Figure was created using the Generic Mapping Tools (GMT) version 6.0.0 (https://www.generic-mapping-tools.org).

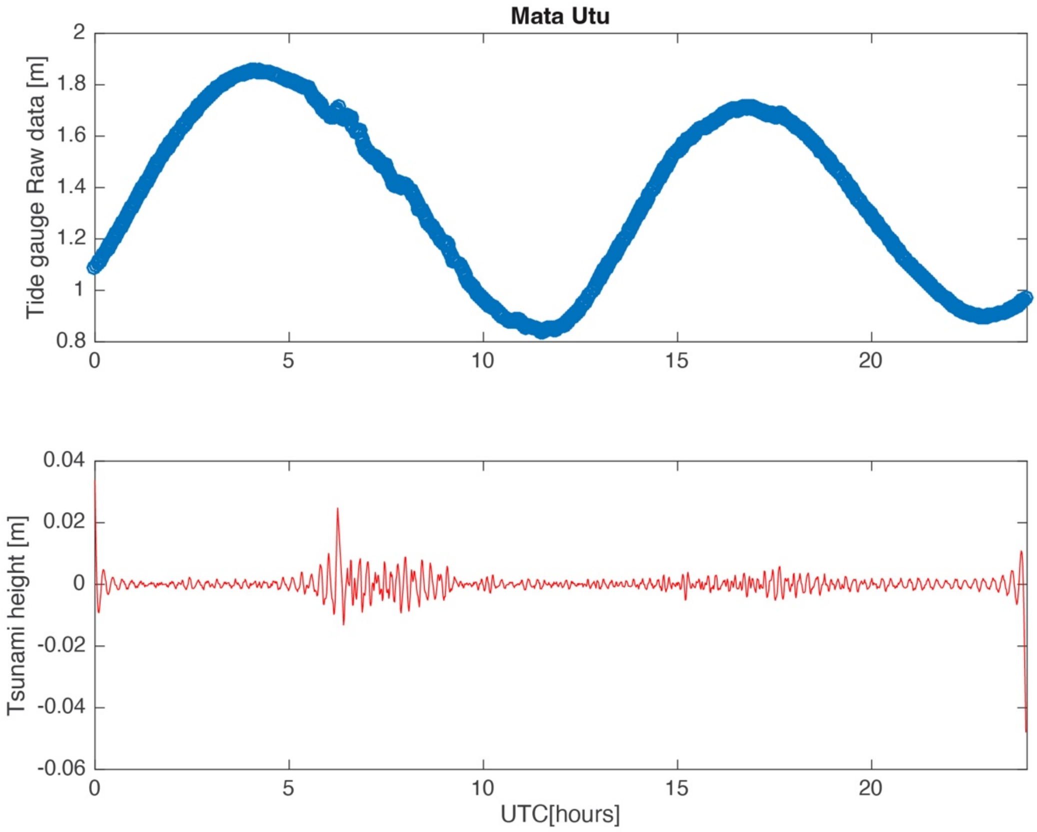

Mata Utu
Tide gauge Raw data [m]
2
1.8
1.6
1.4
1.2
1
0.8
0
5
10
15
20
Tsunami height [m]
0.04
0.02
0
-0.02
-0.04
-0.06
UTC[hours]

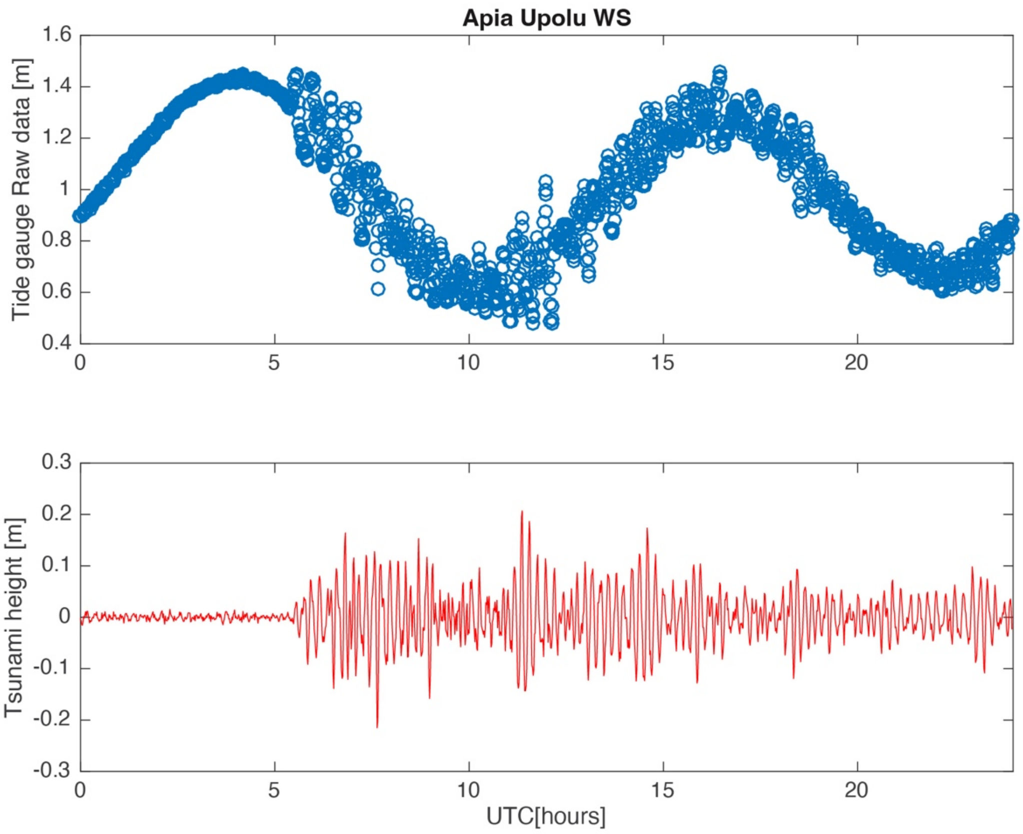
Apia Upolu WS
Tide gauge Raw data [m]
Tsunami height [m]
UTC[hours]

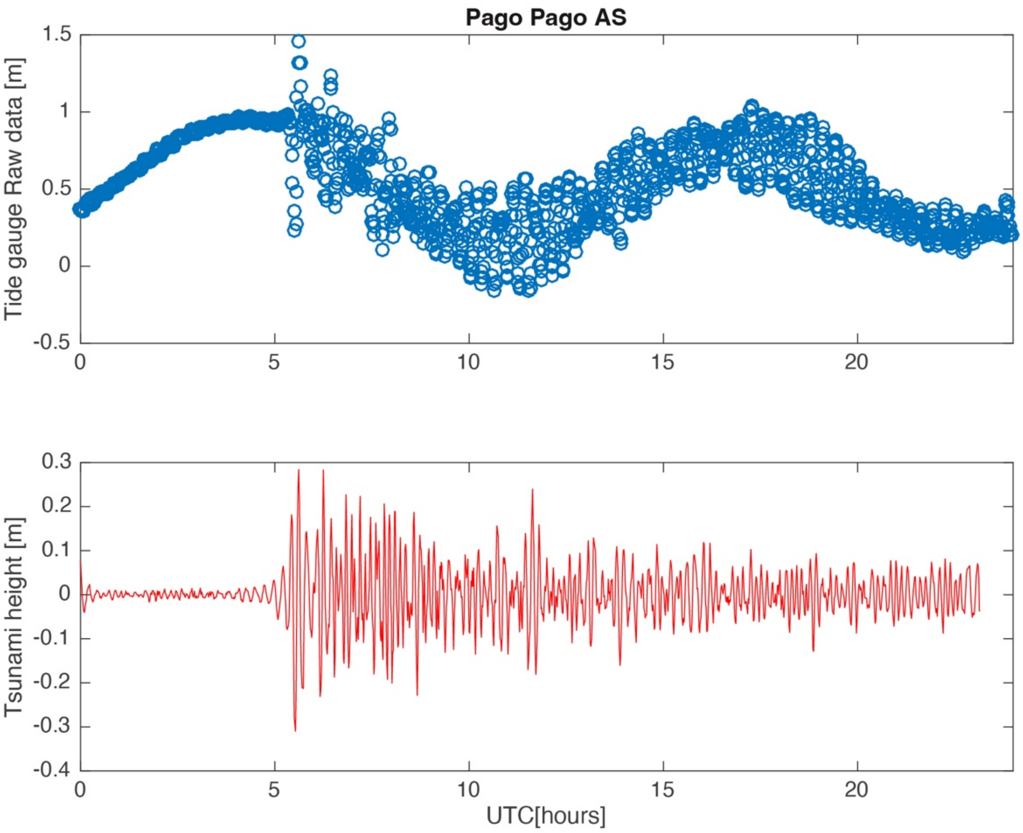
Pago Pago AS
Tide gauge Raw data [m]
Tsunami height [m]
UTC[hours]

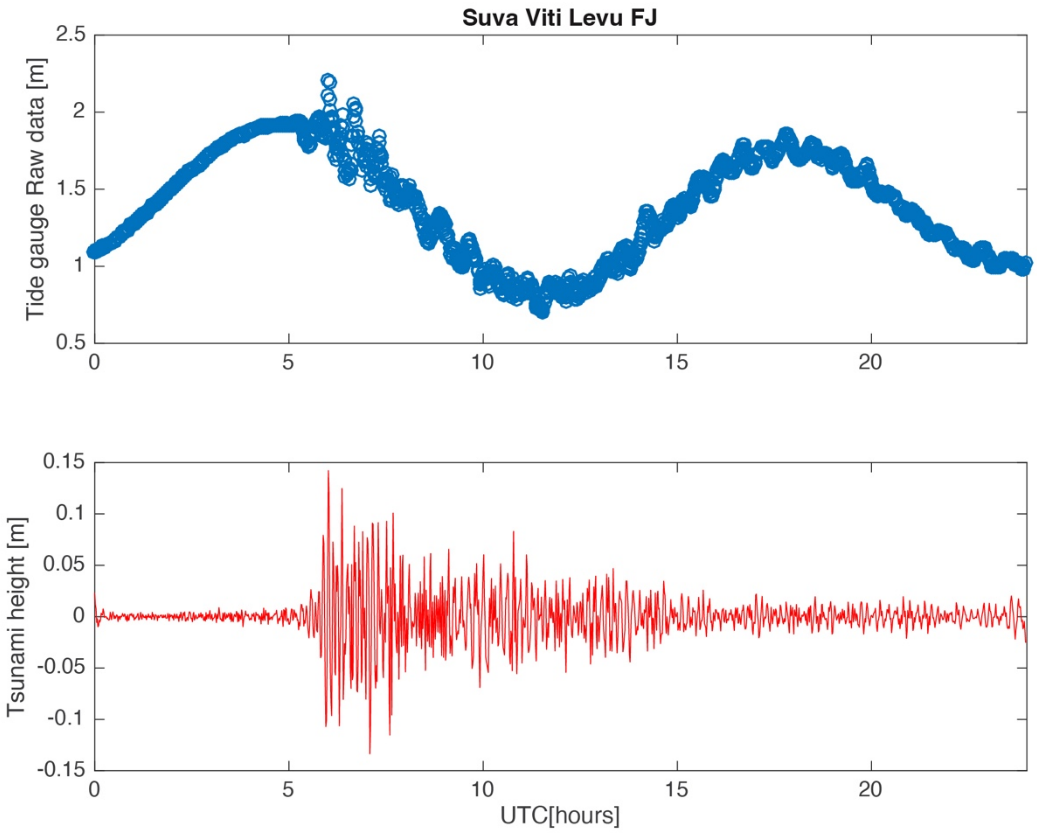
Suva Viti Levu FJ
Tide gauge Raw data [m]
Tsunami height [m]
UTC[hours]

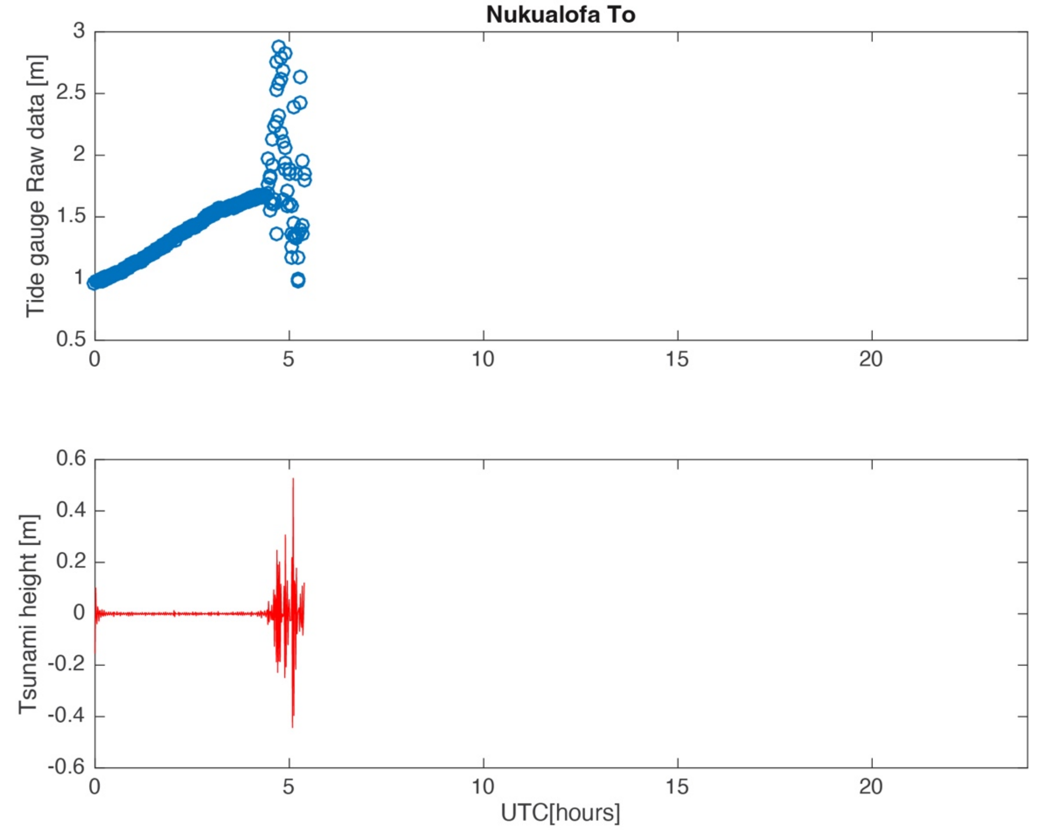
Nukualofa To
Tide gauge Raw data [m]
Tsunami height [m]
UTC[hours]

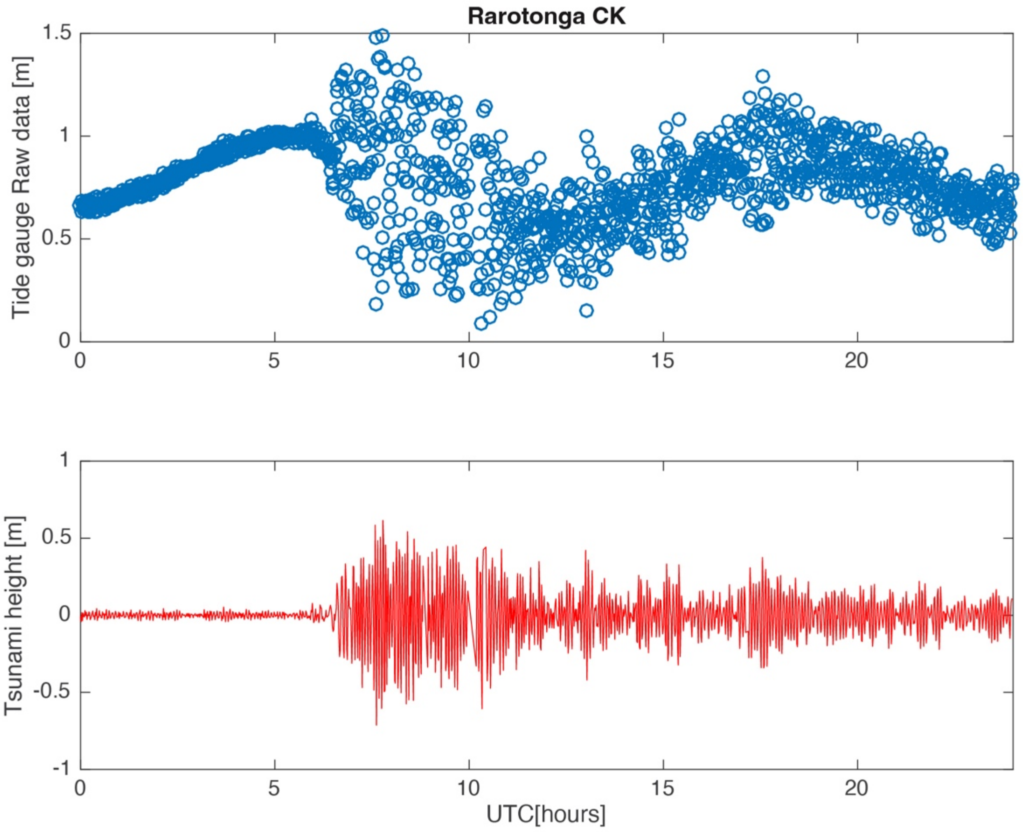
Rarotonga CK
Tide gauge Raw data [m]
1.5
1
0.5
0
0
5
10
15
20
Tsunami height [m]
1
0.5
0
-0.5
-1
0
5
10
15
20
UTC[hours]

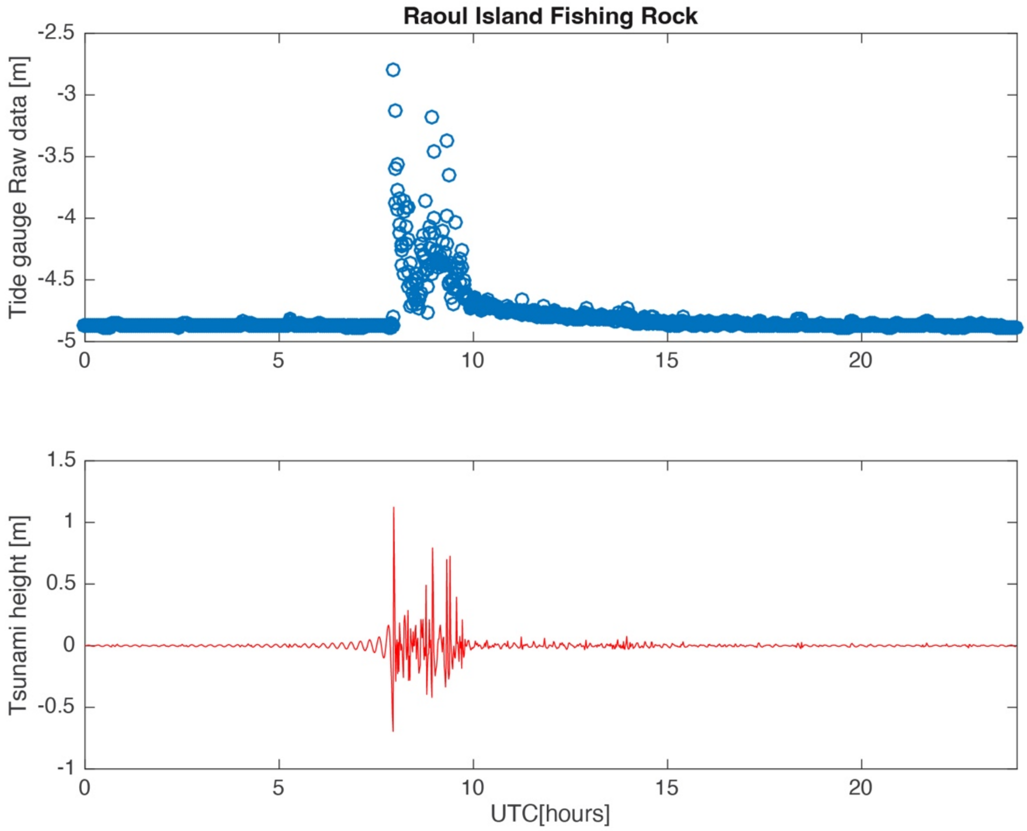
Raoul Island Fishing Rock
Tide gauge Raw data [m]
-2.5
-3
-3.5
-4
-4.5
-5
0
5
10
15
20
Tsunami height [m]
1.5
1
0.5
0
-0.5
-1
0
5
10
15
20
UTC[hours]

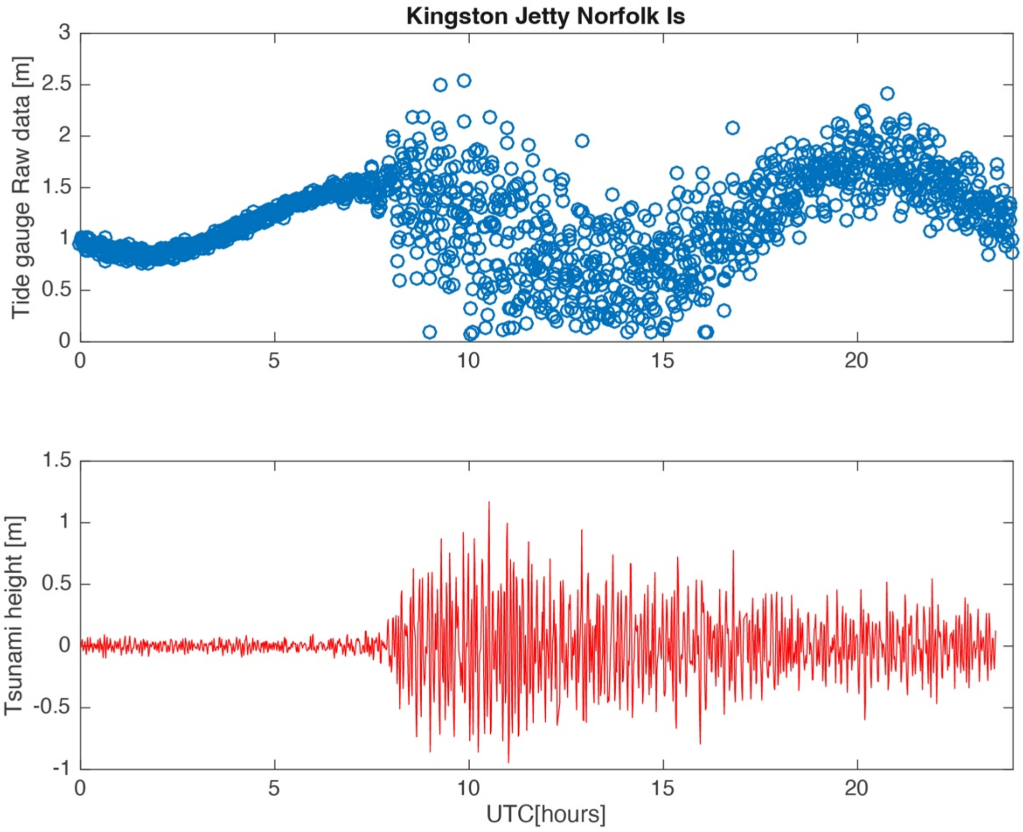
Kingston Jetty Norfolk Is
Tide gauge Raw data [m]
3
2.5
2
1.5
1
0.5
0
0
5
10
15
20
Tsunami height [m]
1.5
1
0.5
0
-0.5
-1
0
5
10
15
20
UTC[hours]

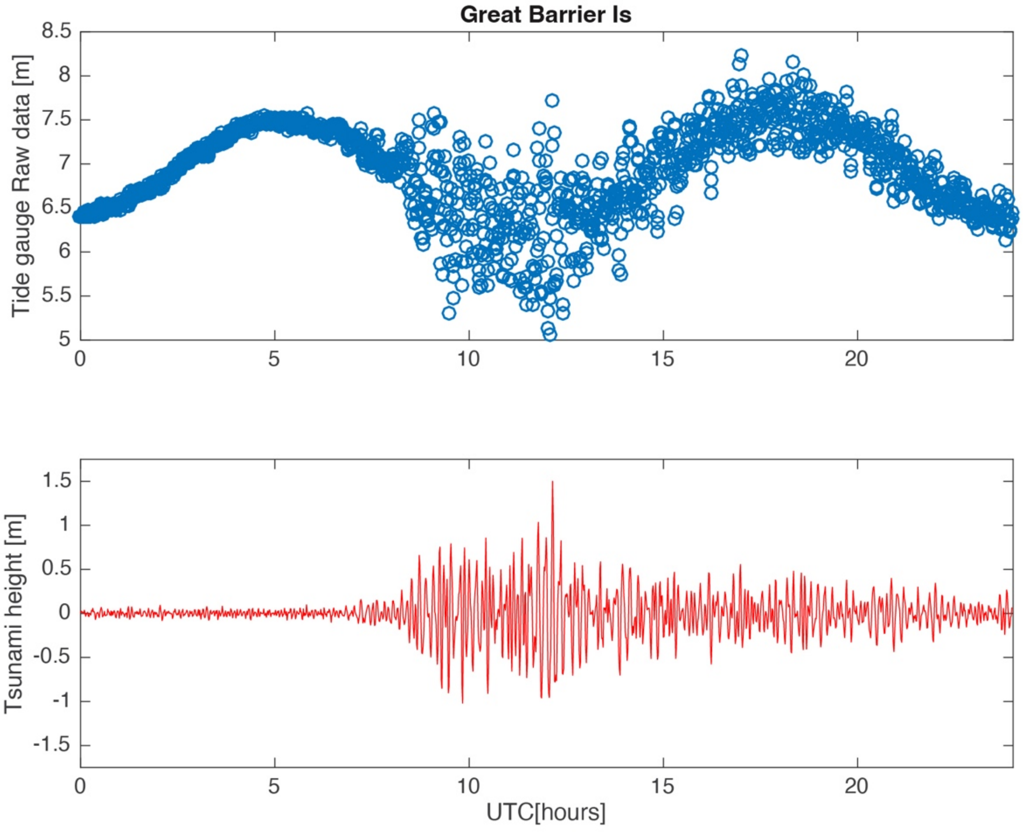
Great Barrier Is
Tide gauge Raw data [m]
8.5
8
7.5
7
6.5
6
5.5
5
0
5
10
15
20
Tsunami height [m]
1.5
1
0.5
0
-0.5
-1
-1.5
0
5
10
15
20
UTC[hours]

Figure S4: Tide gauge sites data in the two panels. First panel represents the raw data of tide gauge sites and second panel represents the filtered signals. The data were filtered using a zero-phase digital high-pass filter to eliminate signals over a 180 min period. This Figure was created using the Generic Mapping Tools (GMT) version 6.0.0 (https://www.generic-mapping-tools.org).
